# Macropinocytosis Is the Principal Uptake Mechanism of Antigen-Presenting Cells for Allergen-Specific Virus-like Nanoparticles

**DOI:** 10.3390/vaccines12070797

**Published:** 2024-07-18

**Authors:** Armin Kraus, Bernhard Kratzer, Al Nasar Ahmed Sehgal, Doris Trapin, Matarr Khan, Nicole Boucheron, Winfried F. Pickl

**Affiliations:** 1 Institute of Immunology, Center for Pathophysiology, Infectiology and Immunology, Medical University of Vienna, 1090 Vienna, Austria; 2 Karl Landsteiner University of Health Sciences, 3500 Krems, Austria

**Keywords:** antigen presenting cells, macropinocytosis, major mugwort pollen allergen, virus-like nanoparticles

## Abstract

Virus-like nanoparticles (VNP) are regarded as efficient vaccination platforms and have proven to be useful for the non-anaphylactogenic delivery of allergen-specific immunotherapy in preclinical models previously. Herein, we sought to determine the mode of VNP uptake by antigen presenting cells (APC). Accordingly, we screened a collection of substances known to inhibit different uptake pathways by APC. The human leukemia monocytic cell line THP-1 and the murine dendritic cell line DC 2.4 were examined for the uptake of fluorescently labelled VNP in the presence or absence of inhibitors. The inhibitory effect of candidate substances that blocked VNP uptake in APC lines was subsequently evaluated in studies with primary APC present in splenocyte and lung cell homogenates in vitro and upon intratracheal application of VNP in vivo. The uptake of allergen-specific VNP in vitro and in vivo was mainly observed by macrophages and CD103^+^ dendritic cells and was sensitive to inhibitors that block macropinocytosis, such as hyperosmolarity induced by sucrose or the polyphenol compound Rottlerin at low micromolar concentrations but not by other inhibitors. Also, T-cell proliferation induced by allergen-specific VNP was significantly reduced by both substances. In contrast, substances that stimulate macropinocytosis, such as Heparin and phorbol myristate acetate (PMA), increased VNP-uptake and may, thus, help modulate allergen-specific T-cell responses. We have identified macropinocytosis as the principal uptake mechanism of APC for allergen-specific VNP in vitro and in vivo, paving the way for further improvement of VNP-based therapies, especially those that can be used for tolerance induction in allergy, in the future.

## 1. Introduction

Enveloped virus-like nanoparticles (VNP) were originally described as non-infectious, sub-cellular, 100–200 nm sized spheres budding from the lipid-raft rich regions of the plasma membrane of producer cells [1], and have, more recently, been shown to be a suitable platform for the targeted insertion of receptor molecules of choice able to deliver signals 1–3 to T cells [2,3,4,5]. Subsequent in vitro and in vivo studies with VNP encasing full length allergen in their virus core led to the suggestion that VNP are being taken up by antigen-presenting cells (APC) in a non-anaphylactogenic manner, leading to the presentation of immunodominant peptides of the VNP-borne allergen to T cells and inducing the differentiation of the latter towards T regulatory cells (Treg) [6,7]. 

The ability of APC to take up VNP has been a curiosity of substantial practical significance, however, the physiological principles governing particle uptake have remained elusive as of yet. In principle, APC have the ability to take up exogenous (foreign) material by several different mechanisms including (i) actin-dependent macropinocytosis [8,9], (ii) clathrin-dependent endocytosis [10,11], (iii) mannose-receptor mediated endocytosis [8,12], (iv) phagocytosis [10,13], (v) phosphatidylserine mediated uptake [14,15], or (vi) Fc-receptor-mediated uptake [16]. Preliminary evidence has suggested that only restricted subsets of splenic and lung APC are equipped with so far undetermined mechanisms to efficiently take up allergen-specific VNP [6]. 

To provide a well-defined screening system for studying VNP uptake by APC, we here established fluorescently labelled VNP and evaluated their binding and uptake by bona fide antigen-presenting cell lines in the presence or absence of a collection of inhibitors with known specificity for distinct uptake pathways. Subsequently, the obtained screening results were corroborated with primary APC types such as those present within mouse splenocytes and lung homogenates. The consequences of pathway inhibition were evaluated in vivo by studying VNP uptake and their inhibition by lung-resident APC and in cell proliferation assays with allergen-specific T cells. Our results reveal that Rottlerin and hyperosmolar sucrose, inhibitors targeting macropinocytosis, significantly restricted the uptake of VNP by APC both in vitro and in vivo. Collectively, the data presented here clearly demonstrate that macropinocytosis represents the principal uptake mechanism for allergen-specific VNP by APC, opening new avenues for studying their facilitated uptake, which may help to shape the tolerogenic potential of allergen-laden VNP in the future.

## 2. Methods

### 2.1. Cell Lines

Human embryonic kidney (HEK-293T) cells (ATCC, Manassas, VA, USA) were cultured in IMDM (Sigma Aldrich, St. Louis, MO, USA) plus 10% FBS (Gibco Life Technologies, Carlsbad, CA, USA) plus 15 mg/L gentamycin (Gibco Life Technologies, Carlsbad, CA, USA). DC 2.4 murine dendritic cells [17] and THP-1 human leukemic cells (ATCC, Manassas, VA, USA) were cultured in RPMI1640 (HyClone Laboratories, Logan, UT, USA) plus 10% FBS plus 15 mg/L gentamycin.

### 2.2. Production of Virus-like Nanoparticles (VNP)

VNP were produced in HEK-293T cells as previously described [6]. Briefly, 3 × 10^6^ HEK-293T cells were seeded on 15 cm cell culture dishes (Sarstedt, Nümbrecht, Germany) and the transfection was performed with 150 μg of MoMLV original gag-pol (OGP) plasmid [18] and 150 μg of pK12_MA::Art v 1. To harvest the VNP, the cell culture supernatant was filtered after 72 h (0.45 μm, Millipore, Billerica, MA, USA) and concentrated by further filtration (Centricon Plus-70, Merck Millipore Ltd., Tullagreen, Ireland) followed by three cycles of ultracentrifugation (SW41 Ti rotor at 1 × 10^5^ g, 1 h, Beckman-Optima LE-80K, Beckman Instruments, Palo Alto, CA, USA). The total protein concentration of the purified VNP was determined with the help of a BCA assay (Micro BCA, Thermo Fisher, Waltham, MA, USA). Previously, it has been proven that VNP can be stored at 4 °C for up to 4 weeks, without influencing their biological activity [6].

### 2.3. Generation of Fluorescently Labelled Virus-like Nanoparticles (VNP)

For the generation of FITC labelled VNP, 100 µg of MA::Art v 1 VNP (1mg/mL) were incubated, in the dark at room temperature for 20 min, with 50 μg of NHS-fluorescein (Thermo Scientific, Waltham, MA, USA) in a NaHCO_3_ (Sigma Aldrich, St. Louis, MO, USA) buffer (pH 8.5). To generate cell mask orange (CMO) labelled VNP, 100 µg of MA::Art v 1 VNP (1 mg/mL) were incubated, in the dark on ice for 10 min, with 1× of CMO membrane dye (1000× solution; Thermo Scientific, Waltham, MA, USA) in a volume of 100 µL. To remove free fluorophores, the VNP preparations were dialyzed against PBS (Ca^2+^ and Mg^2+^) (Gibco Life Technologies, Carlsbad, CA, USA), using a microdialysis block (System 500 Microdialyser; Pierce, Rockford, IL, USA). The dialysis was carried out in two steps against 5 L PBS each, with the total dialysis time being 72 h. The clean labelled particles were stored protected from light at 4 °C.

### 2.4. Flow Cytometry-Based Binding and Uptake Studies with Cell Lines

The uptake of FITC-labelled MA::Art v 1 VNPs and the macropinocytosis marker lucifer yellow [8] by THP-1 (ATCC, Manassas, VA, USA) and DC 2.4 cells [17] was investigated in the presence or absence of selective uptake inhibitors (Table 1). The constitutive uptake was determined in the presence of FACS buffer [PBS (Gibco Life Technologies, Carlsbad, CA, USA); 0.5% BSA; (Sigma Aldrich, St. Louis, MO, USA)]. The incubation mixture for each 5 mL polystyrene tube consisted of 1.25 × 10^6^ cells/mL and 10 µg/mL of fluorescently labelled VNP or 93.8 μg/mL of lucifer yellow in a total volume of 85 µL. The incubation was carried out for each condition at either 4 °C or 37 °C for 60 min. The 4 °C incubation condition was used as a control for surface binding without active uptake, while the 37 °C condition was regarded as optimal for VNP uptake. After the incubation, 20 μL trypan blue (Lonza, Walkersville, MD, USA) (0.4%) was added to each mixture in order to quench the extracellular fluorescence signal of surface bound FITC-VNP or lucifer yellow [19,20]. Next, the cells were washed twice with FACS washing buffer [PBS (Gibco Life Technologies, Carlsbad, CA, USA); 0.5% BSA (Sigma Aldrich, St. Louis, MO, USA); 0.05% NaN_3_ (Sigma Aldrich, St. Louis, MO, USA); 1 mM EDTA (Sigma Aldrich, St. Louis, MO, USA)]. Before the acquisition, 20 μL DAPI (Sigma Aldrich, St. Louis, MO, USA) (0.125 ng/mL) was added to each tube to facilitate live/dead cell discrimination. The short-term storage until the flow cytometric measurement as well as the transport of cells took place strictly on ice. For sample acquisition, a LSR Fortessa flow cytometer (BD Biosciences, San Jose, CA, USA) equipped with four laser lines was used and the data analysis was performed using the FlowJo analysis software 10.9.0 (BD Biosciences, San Jose, CA, USA). 

### 2.5. Mice

Age-matched B57BL/6J mice (6–10 weeks old) homozygous for an Art v 1_25–36_-specific TCR and HLA-DRA*01/-DRB1*01 heterodimers (TCR-DR1) were used [52]. Mice were regularly monitored for their health status according to the FELASA 2014 recommendations and were found to be free of mouse pathogenic viruses, bacteria, and parasites [53]. All experimental procedures were reviewed and approved by the Institutional Review Board of the Medical University of Vienna and approved by the Federal Ministry of Education, Science and Research, Austria (GZ: 2020-0.223.953; BMBWF-66.009/0234-V/3b/2019; BMWFW-66.009/0161-WF/V3b/2016).

### 2.6. Preparation of Single Cell Suspensions from Lung and Spleen

Mice were sacrificed by cervical dislocation, and lungs and spleen were excised under sterile conditions. Lungs were then cut into 10 mm^3^ pieces which were subsequently incubated with RPMI1640 medium containing DNAse (0.05 mg/mL; Sigma Aldrich, St. Louis, MO, USA) and Liberase TL (0.05 mg/mL; Sigma Aldrich, St. Louis, MO, USA) at 37 °C for 1 h. Subsequently, single cell suspensions of both lungs and spleen were generated by passing the cells through a 70 µm nylon cell strainer (Corning, Corning, NY, USA) followed by collection of cells by centrifugation at 500× *g* for 5 min. Lysis of erythrocytes was achieved by resuspending the cell pellet with ammonium chloride lysis buffer [155 mM ammonium chloride (Merck, Darmstadt, Germany); 10 mM potassium hydrogen carbonate (Merck, Darmstadt, Germany); and 0.1 mM EDTA (Sigma-Aldrich, St. Louis, MO, USA)] at room temperature for 3 min. In the last step, the cells were washed twice with RPMI1640 (HyClone Laboratories, Logan, UT, USA) plus 10% FBS (Gibco Life Technologies, Carlsbad, CA, USA). 

### 2.7. Analyses of CMO-VNP In Vitro Uptake by Primary APC Types

The cells were preincubated with FACS buffer (PBS (Gibco Life Technologies, Carlsbad, CA, USA); 0.5% BSA; (Sigma Aldrich, St. Louis, MO, USA)) alone or in the presence of selective uptake inhibitors (Table 1) at 4 °C for 1 h. Subsequently, the different cell suspensions (1 × 10^6^) were incubated with CMO-VNP (10 µg/cell sample) at 4 °C and 37 °C for 1 h to differentiate between mere binding and bona fide uptake of VNP. 

### 2.8. Determination of CMO-VNP Uptake In Vivo

Mice were anesthetized using a ketamine–xylazine mixture (13.6 mg/mL ketamine (Pfizer, Vienna, Austria); 2.1 mg/mL xylazine (Bayer, Leverkusen, Germany); 7.5 µL per g body weight) for in vivo experiments, and challenged intratracheally with 40 µL of fluorescently labelled MA::Art v 1 VNP (1 mg/mL) under visual control using an otoscope (HEINE Optotechnik, Gilching, Germany). After 24 h, mice were sacrificed by cervical dislocation. Subsequentially, mouse lungs were excised under sterile conditions and single cell suspensions were prepared as described above. 

### 2.9. Analyzing Organ Homogenates after In Vivo or In Vitro Uptake 

Following the uptake step, the cells were washed twice with PBS, then incubated with Aqua Zombie viability dye (Biolegend, San Diego, CA, USA) at room temperature for 10 min and with FcBlock (Biolegend, San Diego, CA, USA) at room temperature for 5 min. To differentiate subsets of splenic and lung APC, cells were then stained with the antibodies listed in Table S1 at 4 °C for 30 min and washed twice with FACS washing buffer [PBS (Gibco Life Technologies, Carlsbad, CA, USA); 0.5% BSA (Sigma Aldrich, St. Louis, MO, USA); 0.05% NaN_3_ (Sigma Aldrich, St. Louis, MO, USA); 1 mM EDTA (Sigma Aldrich, St. Louis, MO, USA)]. For sample acquisition, a LSR Fortessa flow cytometer (BD Biosciences, San Jose, CA, USA) equipped with four laser lines was used and the data analysis was performed using the Flowjo analysis software 10.9.0 (BD Biosciences, San Jose, CA, USA).

### 2.10. Proliferation Assays 

Spleens were excised from TCR/DR1 mice and passed through a 70 µm cell strainer (Corning, Corning, NY, USA) to create single cell suspensions, as per the protocol described above. Splenocyte single cell suspensions (2 × 10^5^/well) were incubated with the indicated stimuli in the presence or absence of the selected uptake-inhibitors (Table 1) in 96-well round bottom plates (Sarstedt, Nümbrecht, Germany). The incubation was performed with 10 µg/mL of allergen-expressing VNP, 10 µg/mL of empty VNP, 1 mM of Art v 1_23–36_ peptide (Proimmune, Oxford, UK), 0.5 µg/mL of rArt v 1 protein (kindly provided by Gabriele Gadermaier, Univ. Salzburg), 4 µg/mL PHA (Sigma Aldrich, St. Louis, MO, USA), 100 nM PMA (Sigma Aldrich, St. Louis, MO, USA), 0.1 µg/mL Ionomycin (Sigma Aldrich, St. Louis, MO, USA) or medium alone, all used in a total volume of 200 µL. 72 h after the incubation with stimuli, the cells were pulsed with methyl-[3H] thymidine (1 µCi/well) for 18 h, harvested and the T-cell proliferation was then quantified on a Beta Counter (Perkin Elmer, Waltham, MA, USA).

### 2.11. Statistical Methods

Groups with normal distribution were tested using one-way ANOVA followed by Holm-Sidak correction for multiple comparisons. If data were not normally distributed, Kruskal–Wallis test was applied followed by Dunn’s multiple comparison correction in between groups. *p* values below 0.05 were considered statistically significant and are denoted as * *p* < 0.05; ** *p* < 0.01; *** *p* < 0.001. The analyses were performed using the GraphPad version 9.0 and 10.0 (GraphPad Software Inc., La Jolla, CA, USA).

## 3. Results

### 3.1. Macropinocytosis Inhibitors Block the Major Uptake of FITC-VNP by Antigen Presenting Cell Lines 

**Figure 1:**
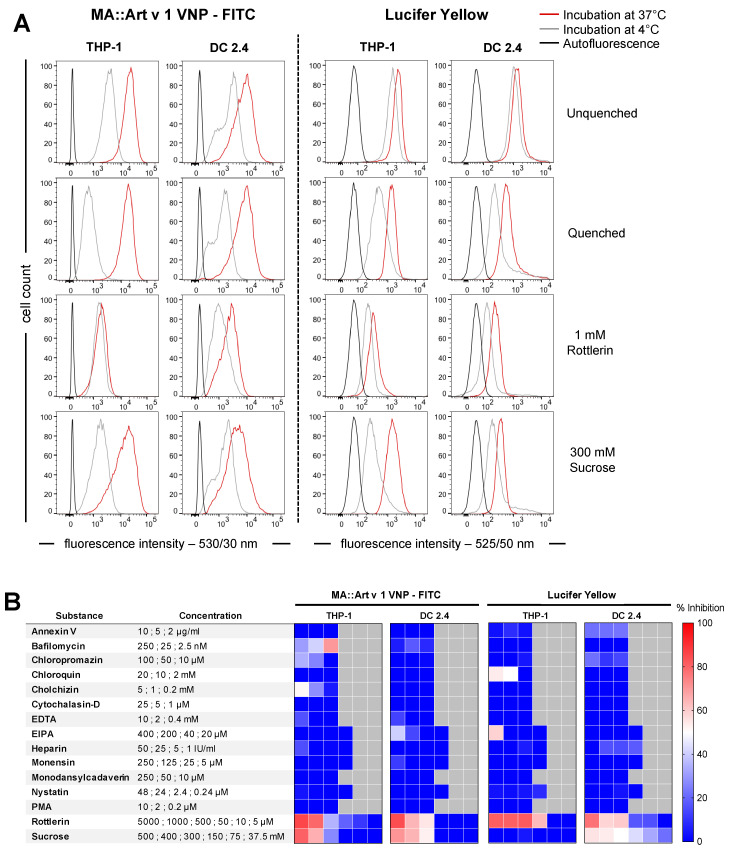
Binding and uptake of FITC-VNP by antigen presenting cell lines. (**A**) Shown are representative overlay histograms of THP-1 and DC 2.4 cell lines incubated with FITC-MA::Art v 1-VNP (left panel) or the macropinocytosis marker lucifer yellow (LY) (right panel) in the presence or absence of selected inhibitors. Black lines indicate autofluorescence in the respective channel, grey lines indicate binding at 4 °C and red lines indicate active uptake at 37 °C. The cell suspensions were incubated with 10 μg/mL of FITC-VNP or 93.5 μg/mL of LY in the absence (rows 1–2) or presence of 1 mM Rottlerin (row 3) or 300 mM sucrose (row 4). *Y*-axes show the cell number and *x*-axes show the fluorescence intensity. Quenching of surface bound fluorescence was achieved by Trypan Blue. For the data shown, at least 2 × 10^4^ single cells were acquired of each sample. (**B**) Heatmap depicting the summary of results obtained with all 15 inhibitors tested at the indicated concentrations. The mean values of inhibition shown are calculated on the basis of the uninhibited controls and are derived from two independently performed experiments, each consisting of duplicates.

VNP have been widely accepted as platforms for the delivery of T-cell antigens in the form of presented peptides or full-length proteins in the past [3,4,6]. While the uptake and presentation of VNP delivered antigens is undisputed, the exact uptake pathway(s) of antigen-presenting cells for VNP has remained elusive as of yet, which precludes more precise steering and modulation of the underlying process. Accordingly, we here applied NHS-chemistry to covalently couple FITC to VNP and used the such established fluorescent VNP to determine the pathways by which they are taken up by the human monocytic THP-1 and the murine dendritic DC 2.4 antigen presenting cell lines. FITC-VNP uptake was studied in the presence or absence of a panel of 15 different inhibitors, known to block different uptake pathways of APC (Table 1). To provide an accurate measure for FITC-VNP uptake and to differentiate it from mere surface binding, we determined the fluorescence signals of APC by flow cytometry upon incubation with FITC-VNP at 4 °C or at 37 °C (red histograms) in the presence or absence of trypan blue, used as a quencher for the fluorescence signal of putatively surface-bound VNP [19,20]. Figure 1A, left panel shows that the (auto)fluorescence of THP-1 cells (gMFI of 103 ± 12 (black histograms)) increased upon co-incubation with FITC-VNP at 4 °C for 60 min to a gMFI of 4515 ± 470 (*p* = 0.0103) (grey histograms) and reached a maximal gMFI of 18,842 ± 245 (*p* < 0.0001) after incubation at 37 °C for 60 min (red histograms). However, a large fraction of the fluorescent signal associated with THP-1 cells after their incubation with FITC-VNP at 4 °C could be quenched by the addition of trypan blue, reducing the gMFI to 2806 ± 417 (*p* = 0.0226), and, thus, could be assigned to surface binding but not the internalization of FITC-VNP to THP-1 cells. In contrast, after the 37 °C incubation step, the fluorescence signal intensity remained almost unaffected by the quencher (gMFI 18,842 ± 245 non-quenched versus 16,831 ± 258 quenched; (*p* = 0.7850)), indicating that the FITC-VNP were quantitatively taken up by the THP-1 cells at 37 °C. This distinction made it possible to calculate the actual net uptake of FITC-VNP at the physiological temperature of 37 °C (net FITC-VNP uptake = (gMFI at 37 °C with quenching) minus (gMFI at 4 °C with quenching)). 

In experiments designed to determine the pathway responsible for VNP uptake, we noted that the two macropinocytosis inhibitors, Rottlerin and hyperosmolar sucrose, dose-dependently inhibited FITC-VNP uptake by live THP-1 (by 79.6 ± 5.2%, (*p* = 0.0275); and 65.5 ± 8.6%, (*p* = 0.0479); respectively) and DC 2.4 (by 64.3 ± 3.3%, (*p* = 0.0624); and 53.0 ± 13.2%, (*p* = 0.6467); respectively) cells (Figure 1B, left panels and Figures S1 and S2). Of the other 13 pathway inhibitors, only Bafilomycin showed moderate inhibition in THP-1 without toxicity. Bafilomycin (in DC 2.4), Chlorpromazin, Colchizin, and EIPA (both cell lines) showed moderate inhibitory effects, which were, however, paralleled by clear cytotoxicity (Figure S3). Notably, Rottlerin and hyperosmolar sucrose were not toxic at the concentrations used throughout this study, while this was the case for other substances used at high concentrations, such as Monodansylcadaverin (Figure S3).

The observation that Rottlerin, and to a certain degree, also hyperosmolar sucrose inhibited the fluorescence signal obtained upon incubation of APC with FITC-VNP at 4 °C allows the notion that even at this low temperature, macropinocytosis may be active in these cell lines and causes at least uptake of FITC-VNP to some degree, which cannot be controlled entirely by the low temperature alone. Subsequent control experiments performed with lucifer yellow, an independent fluorescent marker for macropinocytosis [8], whose uptake was clearly inhibitable by Rottlerin, substantiated the specific uptake inhibition observed for FITC-VNP by Rottlerin, while hyperosmolar sucrose inhibited LY uptake exclusively in DC 2.4 cells (Figure 1A,B, right panels and Figures S4 and S5). Additionally, we used FITC-Dextran as a second uptake tracer to control for the specificity of inhibitors. FITC-Dextran had been reported to be taken up via mannose receptors [8], which are expressed on DC 2.4 but not on THP-1 cells. Notably, Rottlerin did not affect the uptake of FITC-Dextran, while it inhibited LY uptake, further proving the specificity of Rottlerin as a selective macropinocytosis inhibitor. Moreover, complementary experiments performed with a split luciferase reporter cell system, in which an enzymatically-deficient form of nano-luciferase (LgBiT) was expressed in the APC lines, which were then incubated with complementation-proficient peptide (referred to as HiBiT)-containing allergen-laden VNP, corroborated the clear-cut inhibition of VNP uptake by hyperosmolar sucrose in both DC 2.4 and THP-1 cells.

While the above supports the view that APC lines take up VNP mainly by macropinocytosis, we next sought to investigate whether this holds true for primary APC as well. Therefore, we turned to experiments with spleen and lung APC in vitro followed by the evaluation of VNP uptake by lung APC in vivo.

### 3.2. Primary APC Take Up VNP by Macropinocytosis

To corroborate the results obtained with APC lines, we conducted uptake studies with fluorescent MA::Art v 1 VNP in primary lung and spleen cell cultures of double transgenic mugwort pollen allergy mice [52] followed by multi-color flow cytometry for the identification of involved APC subsets [54]. 

Preliminary experiments performed with FITC-VNP did not reveal appreciable fluorescence signals with primary APC, which was most likely due to the acidic pH-dependent, intracellular quenching of the FITC fluorescence in the endocytotic compartments of APC [55]. Therefore, we here switched to the use of cell mask orange labelled (CMO)-VNP as described previously [6], which proved to be more suitable for studies with primary APC types.

**Figure 2:**
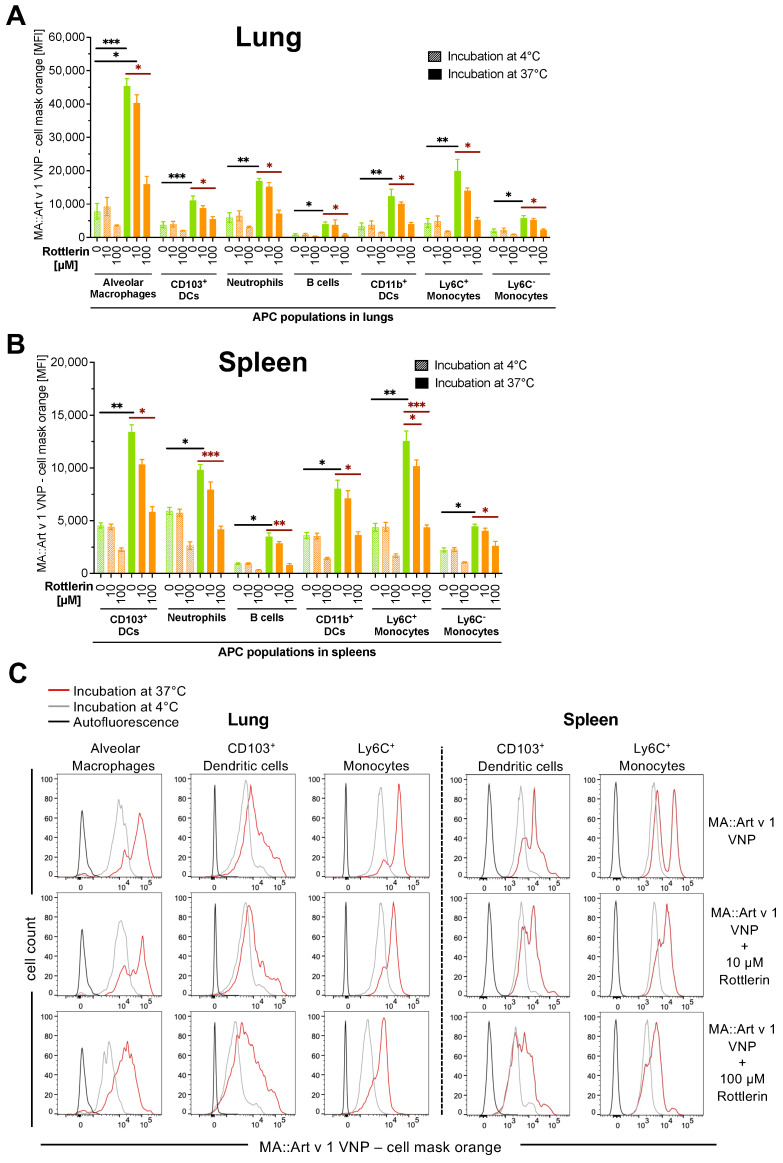
Binding and uptake of fluorescent CMO-VNP by primary antigen presenting cells. Shown is the summary (two independently performed experiments with two mice per condition) of the binding and uptake of cell mask orange labelled (CMO)-MA::Art v 1-VNP (10 μg/mL) (measured as mean fluorescence intensity, *y*-axes) by the indicated APC populations (*x*-axes) in lung (**A**) and spleen (**B**) cell homogenates, in the presence (orange bars) or absence (green bars) of Rottlerin. Hatched bars indicate binding at 4 °C and closed bars indicate active uptake at 37 °C. Significant differences are indicated in black for the uptake of VNP (comparison between 4 °C and 37 °C condition) and in red for the inhibition of VNP uptake (37 °C, with and without Rottlerin). (**C**) Representative overlay histograms for APC types that actively take up VNP present within lung (left panel) and spleen (right panel) cell homogenates, in the presence or absence of the indicated concentrations of the macropinocytosis inhibitor Rottlerin. Black lines indicate autofluorescence in the respective channel, grey lines indicate binding at 4 °C and red lines indicate active uptake at 37 °C. For the data shown, at least 2 × 10^5^ single cells were acquired of each sample. Data show the summary of two independent experiments performed in triplicates. Kruskal–Wallis test followed by Dunn’s correction (**A**,**B**). *, *p* < 0.05; **, *p* < 0.01; ***, *p* < 0.001.

In mouse lung cell cultures, the uptake of CMO-VNP after incubation at 37 °C, revealing a signal over the background binding seen at 4 °C, was most remarkable for alveolar macrophages (AM), CD103^+^ DC and Ly6C^+^ monocytes with resulting increases in cellular fluorescence of 5.8 ± 0.3-fold, 4.3 ± 0.6-fold and 5.4 ± 0.2-fold (closed bars), respectively (Figure 2A). The other APC types took up CMO-VNP much less efficiently, resulting in a lower increase of cellular fluorescence (neutrophils, 2.5 ± 0.1-fold; B cells, 3.8 ± 0.6-fold; CD11b^+^ DC, 3.1 ± 0.5-fold; and Ly6C- monocytes, 2.7 ± 0.4-fold). Significantly, the macropinocytosis inhibitor Rottlerin reduced the CMO-VNP uptake by 63.8 ± 3.1% in AM (*p* = 0.0002), by 51.7 ± 8.3% in CD103^+^ DC (*p* = 0.0002) and by 72.8 ± 6.5% in Ly6C^+^ monocytes (*p* = 0.0002) when applied at 100 µM, compared to untreated cells. A 10-fold lower Rottlerin concentration of 10 µM still inhibited VNP uptake by 12.3 ± 3.3% in AM (*p* = 0.1032), by 21.0 ± 6.0% in CD103^+^ DC (*p* = 0.1320) and by 28.7 ± 4.5% in Ly6C^+^ monocytes (*p* = 0.1032), respectively. 

In a similar way, splenocytes were evaluated for CMO-VNP uptake, which essentially confirmed the data obtained with primary lung APC (Figure 2B). In particular, CD103^+^ DC and Ly6C^+^ monocytes showed superior CMO-VNP uptake (>3-fold increase of cellular fluorescence at 37 °C compared to 4 °C) when compared to the other spleen-resident APC types. Again, CMO-VNP uptake could be significantly inhibited by coincubation of splenocytes with 100 µM Rottlerin (Figure 2C), significantly reducing the VNP-uptake by 56.6 ± 8.2% in CD103^+^ DC (*p* = 0.0003) and by 64.4 ± 7.6% in Ly6C^+^ monocytes (*p* = 0.0007) compared to untreated cells. Even a 10-fold lower Rottlerin concentration (10 µM) moderately inhibited VNP uptake by 22.9 ± 9.1% in CD103^+^ DC (*p* = 0.1671) and by 17.5 ± 6.6% in Ly6C^+^ monocytes (*p* = 0.3887), respectively. 

Interestingly, these in vitro studies could not identify a single APC type to be the primary cause for VNP uptake. However, we could confirm the stronger uptake activity of certain cell types. In particular, CD103^+^ DC and Ly6C^+^ monocytes in the lung and spleen as well as AM in the lung were the main APC types responsible for VNP uptake. Notably, Rottlerin did not change the expression levels of MHC class II molecules on the different APC types under investigation (Figure S7A,B). 

### 3.3. The Macropinocytosis Inhibitor Rottlerin Blocks Uptake of Allergen-Laden VNP In Vivo

**Figure 3:**
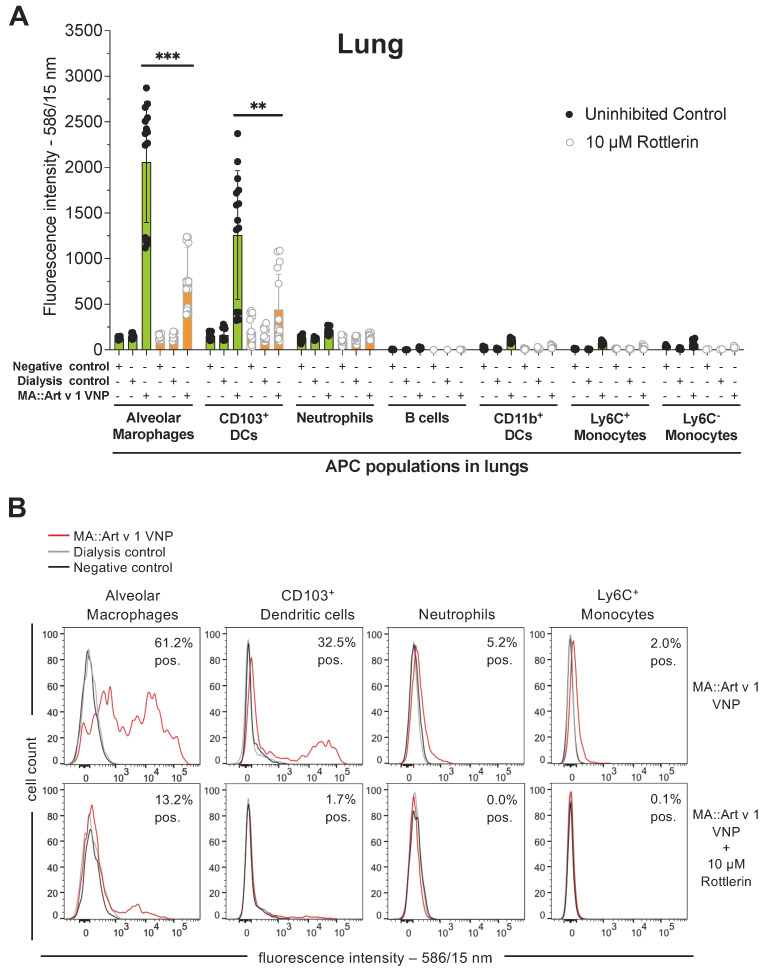
Binding and uptake of fluorescent CMO-VNP by lung resident primary antigen presenting cells in vivo. (**A**) Summary of three independent experiments with three mice per condition evaluating the in vivo uptake of CMO-MA::Art v 1-VNP (*y*-axes) by antigen-presenting cell (APC) populations (*x*-axes) present in the lungs of C57BL/6 allergy mice in vivo. Mice were challenged intratracheally (i.t.) with CMO-MA::Art v 1-VNP, PBS or dialysis control (representing dialyzed PBS-CMO at the concentration used for VNP staining) and rested for 24 h. Subsequently, mice were sacrificed, lungs were surgically removed and lung cell homogenates were evaluated for the indicated APC populations and CMO fluorescence. Green bars show the APC fluorescence in the absence of, and orange bars in the presence of, 10 µM Rottlerin. Kruskal–Wallis test followed by Dunn’s correction (**A**). **, *p* < 0.01; ***, *p* < 0.001. (**B**) Representative overlay histograms of selected lung APC populations of mice which were challenged with 10 μg/mL of CMO-VNP (red lines), PBS (black lines) or dialysis control (grey lines). For the data shown, at least 2 × 10^5^ single cells were acquired of each sample.

Next, we determined whether, also, in vivo macropinocytosis is the preferred uptake mechanism for VNP by APC. For that purpose, double transgenic mugwort pollen allergy mice (C57BL/6J background) were challenged with CMO-labelled MA::Art v 1 VNP intratracheally and CMO-VNP uptake by lung resident APC was studied 24 h later. Notably, AM and CD103^+^ DC were the only APC to show substantial uptake of CMO-VNP, as revealed by their significant increase in cellular fluorescence compared to the PBS negative controls (gMFI 129 ± 12 (AM), 150 ± 37 (CD103^+^ DC), 123 ± 28 (neutrophils), 0 ± 19 (Ly6C^+^ monocytes)) resulting in gMFI values of 2058 ± 662 (AM) and 1257 ± 707 (CD103^+^ DC), respectively. (Figure 3A). Other cell types, such as neutrophils (gMFI of 216 ± 34) and Ly6C^+^ monocytes (gMFI of 74 ± 21), did not show significant VNP uptake over background. These results confirmed the above data obtained with lung cells in vitro and showed that indeed, AM and CD103^+^ DC are the main APC types responsible for VNP uptake in vivo. Co-application of the macropinocytosis inhibitor Rottlerin at 10 µM, significantly reduced the CMO-VNP uptake of AM by 62.0 ± 16.7% (*p* = 0.0002) and of CD103^+^ DC by 65.0 ± 30.8% (*p* = 0.0046) to gMFI levels of 783 ± 344 and 440 ± 388, respectively. The overlay histograms in Figure 3B show that significant fractions of AM and CD103^+^ DC have taken up the fluorescent VNP when compared to the negative controls. Co-application of Rottlerin significantly reduced the CMO^+^ APC populations from 61.2 ± 9.8% to 13.2 ± 7.4% (*p* = 0.0204) and from 32.5 ± 5.3% to 1.7 ± 1.3% (*p* = 0.0300), respectively. No significant CMO^+^ fluorescent populations were detected among other APC types, as shown for neutrophils and Ly6C^+^ monocytes (Figure 3B). Notably, Rottlerin treatment did not change the expression levels of MHC class II molecules on the different APC types (Figure S7C,D). 

### 3.4. Inhibition of Macropinocytosis in Antigen-Presenting Cells Co-Incubated with Allergen-Laden VNP Reduces Their T-Cell Activating Properties

**Figure 4:**
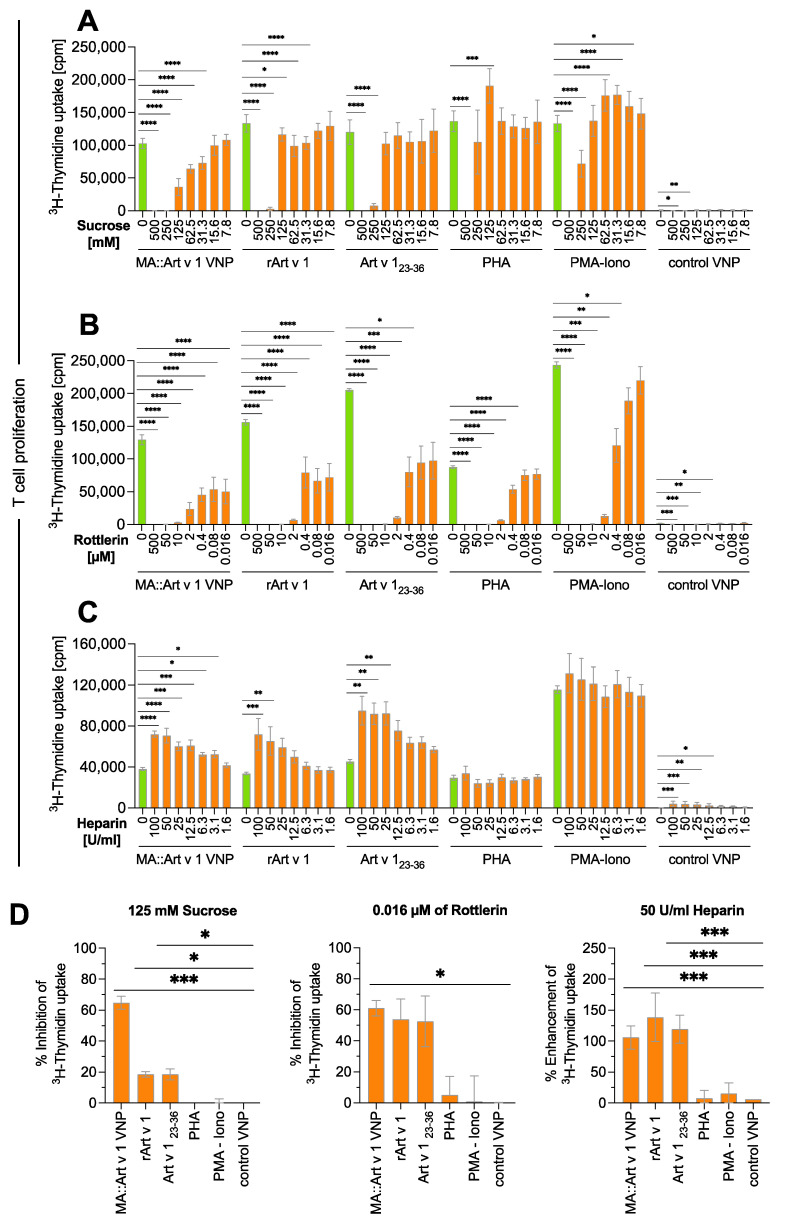
Inhibition of macropinocytosis reduces while activation of macropinocytosis increases the antigen-presenting capacity of splenic APC. Shown is the influence of hyperosmolar sucrose (**A**), Rottlerin (**B**), and Heparin (**C**) on MA::Art v 1-VNP-induced proliferation of splenocytes derived from allergy mice. Green bars show the proliferation in the absence and orange bars in the presence, of inhibitors or activators. Data (mean + SD) show the summary of three independent experiments performed in triplicates. The *y*-axes show the incorporation of methyl-[3H] thymidine into the cellular DNA (background corrected) as a proxy for cellular proliferation, and the *x*-axes represent the applied stimuli and the respective concentrations of applied inhibitors. The T-cell proliferation was quantified after three days plus 18 h of labelling on a beta counter (Perkin Elmer, Waltham, MA, USA). (**D**) Percent inhibition/enhancement of proliferation induced by the three applied substances in selected concentrations. Data were analyzed by the Kruskal–Wallis test followed by Dunn’s correction. *, *p* < 0.05; **, *p* < 0.01; ***, *p* < 0.001; ****, *p* < 0.0001.

In a final step, we studied the impact of the modulation of macropinocytosis on the VNP-dependent presentation of nominal antigen or the immunodominant Art v 1_23–36_ peptide. For that purpose, we incubated mouse splenocytes from double transgenic mugwort allergy mice with allergen-laden VNP in the presence or absence of macropinocytosis inhibitors. The addition of titrated amounts of the macropinocytosis inhibitor sucrose (range 7.8–500 mM) to such cultures dose dependently reduced the VNP-induced T-cell proliferation, which is taken as a measure of reduced APC uptake and subsequent presentation of the antigen to T cells. Specifically, sucrose (Figure 4A) at a concentration of 125 mM significantly inhibited T-cell proliferation by allergen-laden VNP by 64.8 ± 4.2% (*p* = 0.0001), while it only moderately inhibited rArt v 1 protein-induced (by 18.5 ± 7.6%; (*p* = 0.0256)) and Art v 1_23–36_ peptide-induced (by 18.5 ± 12.3%; (*p* = 0.0130)) T-cell proliferation, compared to untreated cells. Neither polyclonal PHA-induced (*p* > 0.9999) nor PMA/Ionomycin-induced (*p* = >0.9999) T-cell proliferation were affected. Notably, the inhibition of T-cell proliferation induced by MA::Art v 1 VNP but not by control stimuli was clearly evident, also, at the lower sucrose concentrations of 62.5 mM (37.6 ± 6.3% inhibition; (*p* = 0.0120)) and 31.3 mM (29.0 ± 9.2% inhibition; (*p* = 0.0438)), respectively, while sucrose concentrations >125 mM appeared to be T-cell toxic and, thus, the observed reduced levels of T-cell proliferation had to be considered as non-specific. Titrated amounts of Rottlerin significantly inhibited T-cell proliferation induced by allergen-laden VNP. The dose-response experiments in Figure 4B show that Rottlerin at a concentration of 0.016 and 0.08 mM clearly inhibited T-cell proliferation induced by allergen-laden VNP (by 61.1 ± 5.0% at 0.016 mM; (*p* = 0.0398) and by 58.6 ± 6.6% at 0.08 mM; (*p* = 0.0495)), compared to untreated cells. Moreover, it inhibited rArt v 1 (by 53.8 ± 13.1% at 0.016 mM; (*p* = 0.1904) and by 57.3 ± 14.4% at 0.08 mM; (*p* = 0.1017)), and Art v 1_23–36_ peptide induced T-cell proliferation by a similar degree (by 52.5 ± 16.4% at 0.016 mM; (*p* = 0.3261) and by 54.0 ± 18.6% at 0.08 mM; (*p* = 0.1904)), respectively. This was in clear contrast to the almost non-existing inhibition by Rottlerin of the polyclonal T-cell proliferation induced by PHA (5.1 ± 11.9% at 0.016 mM; (*p* = 0.5127) and by 2.1 ± 8.2% at 0.08 mM; (*p* = 0.5270)) and PMA/Ionomycin (by 0.8 ± 16.5% at 0.016 mM; (*p* = 0.7434) and by 7.3 ± 16.7% at 0.08 mM; (*p* = 0.3261)). In order to separate the potential toxicity of Rottlerin on T cells from its action on macropinocytosis in APC, Rottlerin was first pre-incubated with APC, followed by its removal and the subsequent co-incubation of the such pretreated APC with allergen-specific T cells and the different allergen-specific and polyclonal T cell stimuli. These experiments revealed that the effect of Rottlerin (2–50 µM) was limited to the uptake of VNP, but not to that of soluble allergens or the activation by polyclonal stimuli (Figure S8).

In contrast to the macropinocytosis inhibitors, Heparin, a previously described substance for the upregulation of pinocytosis [37], which we also confirmed on primary cells herein (Figure S6 and Supplementary Results), was evaluated for its possibly enhancing effects on the uptake of allergen-laden VNP at a range of concentrations (1.56–100 U/mL). These dose-response experiments show (Figure 4C) that 50 U/mL of Heparin was able to enhance T-cell proliferation induced by allergen-laden VNP (by 105.9 ± 56.3%; (*p* = 0.0005)), rArt v 1 protein (by 138.4 ± 100.5%; (*p* = 0.0003)) and Art v 1_23–36_ peptide (by 119.3 ± 68.1%; (*p* = 0.0002)), respectively (compared to untreated cells). In contrast, T-cell proliferation induced by PHA (by 7.8 ± 38.1%; (*p* > 0.9999)) and PMA/Iono (by 15.3 ± 52.4%; (*p* = 0.6465)) was not significantly enhanced by Heparin. For easy comparison, relative levels of inhibition/enhancement of proliferation induced by 125 mM sucrose, 0.016 µM Rottlerin and 50 U/mL Heparin are shown in Figure 4D. Of note, sucrose-induced inhibition of proliferation appeared to be more specific for allergen-laden VNP, since it inhibited T-cell proliferation induced by soluble allergens only moderately (<20%). This was somewhat different from the Rottlerin-based inhibition of allergen-specific T-cell proliferation, which inhibited VNP-induced proliferation but also the one induced by soluble allergen protein and allergen-peptide to a similar degree.

## 4. Discussion

Enveloped virus-like nanoparticles budding from the lipid raft rich regions of producer cells [1] have proven to be excellent tools for the creation of reductionist antigen presentation platforms [3,4] that can be decorated with molecules of choice [5,56,57] both on their surface but also in their interior. As demonstrated previously, VNP laden with nominal allergen prime T cells in vivo towards a Treg phenotype in a non-anaphylactogenic manner [6]. The practical use as an antigen-delivery shuttle demanded a closer scrutiny of the underlying uptake mechanism for VNP and the primary cell type(s) responsible for it. Accordingly, we here investigated the potential uptake mechanisms of antigen presenting cell (APC) lines and primary murine lung and spleen APC for enveloped allergen-laden VNP generated by expression of *Moloney* murine leukemia virus core sequences. Using specific uptake pathway inhibitors in combination with flow cytometric tracing of fluorescently labelled allergen-specific VNPs, we here show that VNP are most efficiently taken up in vitro and in vivo by alveolar macrophages (AM) and CD103^+^ dendritic cells by a mechanism commonly referred to as macropinocytosis.

The principle activity of cells to take up particulate extracellular material was described by *Ilya Metchnikoff* in 1882 [58]. Subsequently, a number of detailed mechanisms for particle uptake were identified, such as phagocytosis, receptor or clathrin coated pit mediated uptake, phosphatidylserine-mediated uptake, micro- and macropinocytosis. The latter was shown to be initiated by actin-driven extension of >250 nm sized plasma membrane ruffles [59], which appear as phase-bright macropinosomes upon microscopic examination. Macropinocytosis is an important, evolutionary conserved cellular uptake mechanism which enables APC such as DC and macrophages to survey their surroundings for potentially dangerous material (microbes, toxins, accumulating cell detritus, etc.) [8,9]. In addition, macropinocytosis plays an important role in the uptake of nutrients, especially in phases of nutrient restriction, during which ‘macropinocytotic sips’ can rescue starving cells [60]. However, some viral pathogens, such as vaccinia [61], human herpesvirus 8 [62], and HIV-1 [63], hitchhike the macropinocytotic pathway and, thereby, succeed in infecting their target cells. 

Our study demonstrates selective inhibition of *Moloney* VNP uptake upon inhibition of macropinocytosis by Rottlerin and hyperosmolar sucrose but not by other macropinocytosis inhibitors such as cytochalasin-D, amiloride and the lysosomotropic agents chloroquine and bafilomycin [64,65]. In our study, we have tested the specificity of macropinocytosis inhibitors by using the macropinocytosis tracer molecule lucifer yellow (LY), and we confirmed that its uptake was very efficiently inhibited by Rottlerin and hyperosmolar sucrose, while both substances did not inhibit the uptake of FITC-Dextran.

Our findings are in line with those of previous studies demonstrating selective inhibition of macropinocytosis in dendritic cells by Rottlerin but not cytochalasin-D or amiloride [48,49], explaining the lower selectivity of the latter two substances when screening with immortalized cell lines. 

In previous studies, the inhibitory effect of Rottlerin was attributed to the PKC- and PI3K-dependent [66,67] reduction of actin reorganization rather than to the inhibition of membrane ruffles, the former being an essential step during macropinocytosis [48]. Macropinocytosis may be favored by a slow increase of intracellular Ca^2+^, as has been demonstrated for moDC previously [68]. By activating Ca^2+^ activated K^+^ channels, Rottlerin may stimulate Ca^2+^ efflux and, therefore, inhibit macropinocytosis [69,70].

Hyperosmolarity induced by sucrose inhibits the number of plasma invaginations and reduces the net surface area of cells and, thereby, impacts the fluid phase and receptor-mediated endocytosis [50]. Collectively, these data suggest that the herein studied VNP are preferentially taken up by the constitutive form of macropinocytosis, which is dependent on the above-mentioned kinases and elevated intracellular Ca^2+^ levels [71,72].

Macropinosomes are distinct from other possible uptake mechanisms, they show no apparent coat (e.g., clathrin), are heterogenous in their size and with a diameter ranging from 0.2 µm to 5 µm, they are clearly larger than clathrin-coated vesicles (about 0.05 µm up to ≤0.1 µm) for example [73,74]. MA::Art v 1 VNPs, as used in this study, have a size of approximately 0.1–0.2 µm, as previously determined [6] and are, thus, in the optimal size range for efficient uptake by APC into macropinosomes. In addition to the particle size, previous studies have shown that the surface charge of particles influences their uptake, with negatively charged compared to neutral particles being taken up much more efficiently by APC [75,76]. This is consistent with the efficient uptake of MA::Art v 1 VNP by AM and CD103^+^ DC observed herein, for which a zeta potential of −14 mV has been demonstrated previously [6].

Another intriguing finding was the higher sensitivity and selectivity of the Rottlerin-mediated inhibition of VNP uptake in vivo as compared to the in vitro experiments. While inhibition of macropinocytosis by APC lines in vitro required millimolar Rottlerin concentrations, macropinocytosis by primary APC in lung and spleen homogenates could be blocked with one log lower, i.e., 100 µM Rottlerin, concentrations. However, the cell type selectivity of the observed in vitro VNP uptake was not impressive and involved a number of APC. AM and CD103^+^ cells prominently took up VNP, but other APC cell types were also quite efficient in that respect. 

Yet, the cell type selectivity of VNP uptake was significantly different in lung homogenates in vitro compared to mice treated with VNP intratracheally in vivo. This phenomenon may be due to (i) the peculiar anatomical structure of the lung giving the cells surveying the alveolar barrier an advantage (e.g., AMs and migratory CD103^+^ DC); (ii) the presence of higher concentrations of VNP in the confined environment in vitro; (iii) the higher constitutive macropinocytosis of immortalized antigen-presenting cell lines compared to primary cells; and (iv) the absence of particle-clearing mechanisms and, thus, the longer colocalization times of particles with APC in vitro. Notably, in vivo, as little as 10 µM of Rottlerin was required to achieve effective inhibition of VNP uptake by lung APC, a dosage that was similarly effective in other mouse studies previously [49]. 

The reason why the other macropinocytosis inhibitors such as chloroquine, bafilomycin, and monensin did show only very low levels of inhibition of VNP uptake is puzzling and may have to do with the ‘stringency’ of the cell lines used for screening (THP-1 and DC 2.4 cells) and their high level of constitutive macropinocytosis, which may be inhibitable only by the ‘most potent’ substances. An alternative explanation could be that the lysosomotropic pathway may not be of prime relevance for the here studied uptake of VNP. Interestingly, monensin, in our hands, rather blocked receptor-mediated (uptake of FITC dextran particles, but not liquid-phase uptake such as macropinocytosis, a finding which is not unique to the present study but which has been shown already in previous studies [77,78]. The examples given show the heterogeneity of the uptake mechanisms for the different VNP species and illustrate the need for detailed cell biological studies. 

Similarly to the MoMLV-derived VNP studied herein, also VNP derived from rabbit hemorrhagic disease virus (RHDV) [79], comprising the RHDV capsid protein and selected coupled peptides, predominantly rely on macropinocytosis for their uptake, but in contrast, are mainly taken up by CD11c^+^ DC and F4/80^+^ macrophages. However, macropinocytosis is, by far, not the most important uptake mechanism for VNP. For instance, papilloma virus-like particles [80], comprising the HPV major capsid protein of L1 or L2 fused to antigens of choice, are taken up by APC primarily via clathrin-mediated uptake and only to a lesser extent by macropinocytosis [81]. Yet, adeno-associated virus vectors, which are frequently used for gene therapy [82] and have a size of about 20 nm, [83] are preferentially internalized via clathrin-mediated endocytosis. In contrast, microvesicles (MV) released from hypoxia-treated mesenchymal stem cells displaying a diameter of <100 nm, are recognized and taken up by the phosphatidylserine (PS)-dependent receptor on endothelial cells [15].

Apart from the herein described VNP, lipid-enveloped but also naked mRNA vaccines for inducing SARS-CoV-2 or tumor immunity [84] have been shown to become preferentially taken up by human and murine DC through macropinocytosis [49]. Moreover, macropinocytosis also seems to play a role in the propagation of pathogenic proteins in neurodegenerative diseases. For instance, tau aggregates, which act as seeds for intracellular fibrillization in neurodegenerative diseases like Alzheimer’s disease, were found to be internalized by macropinocytosis [85]. Another protein described to be taken up by macropinocytosis is the mutant superoxide dismutase-1, which is linked to familial forms of ALS [86]. The latter findings fit with our observations that the uptake of nominal allergen but also of allergen peptide can be substantially blocked by the macropinocytosis inhibitor Rottlerin studied herein. 

The fact that allergen-laden VNPs were able to stimulate allergen-specific T cells clearly indicated that at least a fraction of VNPs, after being taken up by macropinocytosis and their downstream introduction into endo-/lysosomes, were transferred to the MHC class II loading compartments (MIIC), where the VNP-expressed Art v 1 protein was proteolytically degraded and its peptides transferred and presented on MHC II molecules. Because hyperosmolar sucrose and Rottlerin dose-dependently blocked the uptake of allergen-laden VNP by APC, it was likely that such pretreated APC would have a reduced ability to present full-length allergen to specific T-cells, whereas they should optimally serve as accessory cells after incubation with polyclonal stimuli. Indeed, sucrose at concentrations of <125 mM inhibited T-cell activation by allergen-laden VNP highly significantly by approximately 50% (*p* < 0.0001), while similar sucrose concentrations barely inhibited T-cell activation by the full-length Art v 1 protein, the immunodominant Art v 1_23–36_ peptide, or the polyclonal T-cell activation by PHA and PMA/Ionomycin. In a similar vein, Rottlerin at nanomolar concentrations (16 nM) highly significantly inhibited T-cell activation upon incubation with allergen-laden VNP but not with the polyclonal stimuli PHA and PMA/ionomycin. Interestingly, in these experiments, Rottlerin-mediated inhibition of antigen presentation was not restricted to allergen-laden VNP but also affected the stimulation performed with the full-length Art v 1 protein and the immunodominant Art v 1_23–36_ peptide, which seemed to indicate that hyperosmolar sucrose and Rottlerin affected macropinocytosis in slightly different ways. However, when we pre-incubated APC preparations with Rottlerin followed by its thorough removal before incubating the such pre-treated APC with allergen-laden VNP and allergen-specific T cells, very similar to hyperosmolar sucrose treatment, the inhibition of T cell proliferation was exclusively seen for cultures co-incubated with VNP preparations but not with soluble antigens (full proteins and immunodominant peptides). Thus, Rottlerin, even in very low concentrations, may have an impact on allergen-specific but not on polyclonal T-cell proliferation when not removed from the APC-T cell cultures.

The data presented here demonstrate that inhibition of macropinocytosis is capable of reducing the uptake of allergen-laden VNP, leading to significantly reduced antigen-specific but not polyclonal T-cell proliferation. By staining MHC class II molecules on the different APC populations, we were able to rule out an effect of Rottlerin on MHC class II expression levels, suggesting that it is indeed the reduced uptake of VNPs and not an impairment of the presentation of the VNP-associated antigens that causes the hypo-stimulatory phenotype of Rottlerin-treated APC.

Although our studies show, for the first time, the functional importance of macropinocytosis for the uptake of allergen-laden VNPs, the identified inhibitors are, themselves, not further helpful for studies aiming at the VNP-based differentiation of T cells. However, the herein obtained knowledge will help us to more specifically target the uptake of VNP by APC and, thus, may improve the VNP-based differentiation of T cells.

Accordingly, we here sought to test substances, such as Heparin, which have been previously described to enhance the pinocytotic uptake by phagocytes [37] for their effect on VNP-based T-cell stimulation. Indeed, Heparin at concentrations of 3–100 U/mL significantly augmented T-cell activation induced by allergen-laden VNP. Interestingly, Heparin also increased proliferation induced by nominal allergen and the immunodominant Art v 1_23–36_ peptide, while it had no influence on polyclonal T-cell activation induced by PHA or PMA/Ionomycin. Of note in that context, Heparin has been shown to promote Treg generation in vivo previously, which had been related to its ability to induce IL-2 production in T cells [87]. In addition, Heparin has been shown to lower the tendency of protein aggregation [88,89], which may facilitate VNP uptake. It will be interesting to see whether Heparin also has effects in vivo. Heparin is momentarily applied in vivo as an anti-coagulant (Antib) in case of coagulopathies. Apart from Heparin, other substances may contribute and improve VNP uptake. Along those lines, the cis-aconitic decarboxylase immune-responsive gene 1 (Irg1) and its metabolic product itaconate, which have been shown to inhibit bacterial infection, intracellular viral replication, and inflammation in macrophages, appear to be very interesting candidates since they have recently been shown to promote phagocytosis in macrophages via the Keap1/Nfr2/CD36 pathway [90]. These substances will be subject to additional studies in the future.

This study also has some limitations. The application of PMA, to enhance pinocytotic uptake [47], elicited an enhanced in vitro uptake of VNP by dendritic cell lines and in primary CD103^+^ dendritic cells. However, its influence on antigen-presentation could not be studied in detail because it also induces T-cell activation per se.

The use of fluorescence for studying VNP uptake does not come without certain caveats. During our investigations, we had to switch from FITC-labelled VNP to CMO-labelled VNP. This was due to issues with the FITC fluorescence signal stability in primary cells, which were attributed to a more active endocytosis system of these cells, and, therefore lower pH values (pH 4.5 to 5.5 for immature DC; approx. pH 4.5 for mature DC and macrophages [91]). FITC is highly pH-sensitive due to the instability of fluorescence upon conformational changes. The FITC single anion (approximately pH 4–6) shows a fluctuating fluorescence, while the neutral and cationic form of FITC (pH < 4) loses fluorescence entirely [92].

## 5. Conclusions

In summary, we here have deciphered macropinocytosis as the principal uptake mechanism of antigen-presenting cells (AM and CD103^+^ DC) for allergen-laden Moloney VNP. Furthermore, we have identified Heparin as an enhancer of particle uptake. This knowledge will allow us to better modulate VNP uptake in vivo and to steer resulting T-cell differentiation in the future. Moreover, future research will help to elucidate whether particles taken up via macropinocytosis in the absence of danger signals may improve tolerization and, thus, represent a promising option for allergen-specific immunotherapy. 

## Figures and Tables

**Table 1:** Uptake inhibitors used in this study.

Inhibitors	Affected Pathway	Additional Functions	References
**Annexin V**	Phosphatidylserine- dependent uptake	also interacts with actin	[14,15,21]
**Bafilomycin A**	Macropinocytosis	n.a.	[22,23]
**Chloropromazin**	Clathrin-mediated uptake	n.a.	[24,25]
**Chloroquin**	Macropinocytosis	effect only in phagocytic cells/interferes with uptake pathways	[23,26]
**Cholchizin**	Phagocytosis/Micropinocytosis	n.a.	[27,28]
**Cytochalasin-D**	Endocytosis	non-specific/interferes with many absorption routes	[10,29,30]
**EDTA**	Metal Ion dependent uptake	n.a.	[8,31,32]
**EIPA**	Macropinocytosis	non-specific/inhibits clathrin-mediated uptake	[33,34]
**Heparin**	Clathrin-mediated uptake	possible influence on macropinocytosis	[35,36,37]
**Monensin**	Macropinocytosis	interference with endocytotic transport (e.g., receptor recycling)	[23,38,39]
**Monodansylcadaverin**	Clathrin-mediated uptake	n.a.	[40,41]
**Nystatin**	Caveolin-mediated uptake	n.a.	[27,42,43]
**PMA**	Caveolin-mediated uptake	differential regulation of many absorption pathways	[9,44,45,46,47]
**Rottlerin**	Macropinocytosis	also inhibits protein kinases	[48,49]
**Sucrose**	Macropinocytosis	lipid-raft mediated endocytosis	[50,51]

Table 1 shows all inhibitory substances and their main inhibitory function/affected pathway, possible limitations of the inhibitors and literature refences used within this study. All substances, except Bafilomycin (Santa Cruz Biotechnology, Dallas, TX, USA) and Heparin (Gilvasan Pharma, Vienna, Austria), were received from Sigma Aldrich (St. Louis, MO, USA). n.a., not applicable.

## Data Availability

Data will be made available on request.

## Supplementary Materials

The following supporting information can be downloaded at: https://www.mdpi.com/article/10.3390/vaccines12070797/s1, Table S1: Antibodies used in this study. Figure S1: Inhibition of uptake of FITC-VNP by THP-1. Figure S2: Inhibition of uptake of FITC-VNP by DC 2.4. Figure S3: Change of viability of cell lines in the presence of different amounts of respective inhibitors as detected by flow cytometry. Figure S4: Inhibition of uptake of lucifer yellow by THP-1. Figure S5: Inhibition of uptake of lucifer yellow by DC 2.4. Figure S6: Enhanced uptake of fluorescent CMO-VNP by primary antigen presenting cells in vitro. Figure S7: Rottlerin does not change the MHC class II expression levels on primary antigen presenting cell types in vitro and in vivo. Figure S8: Pre-incubation of APC with Rottlerin restricts its uptake inhibitory effect to allergen-laden VNP.

## Abbreviations

AM	alveolar macrophage
APC	antigen presenting cells
Art v 1	major mugwort pollen allergen Art v 1
BSA	bovine serum albumin
CMO	cell mask orange
DAPI	4,6-diamidino-2-phenylindole
DC	dendritic cell
EDTA	ethylenediamintetraacetic acid
EIPA	5-ethyl-N-isopropyl amiloride
FITC	fluorescein isothiocyanate
HPV	human papillomavirus
Ig	immunoglobulin
Iono	ionomycin
LY	lucifer yellow
MA	matrix protein (p15) of MoMLV
MA::Art v 1 VNP	VNP expressing shielded Art v 1
mAb	monoclonal antibody
MHC	major histocompatibility complex
MoMLV	Moloney murine leukemia virus
MV	microvesicles
PBMC	peripheral blood mononuclear cells
PBS	phosphate-buffered saline
PHA	phytohemagglutinin
PI3K	phosphatidylinositol-3 kinase
PKC	protein kinase C
PMA	phorbol 12-myristate 13-acetate
PS	phosphatidyl serine
rArt v 1	recombinant Art v 1
RHDV	rabbit hemorrhagic disease virus
SD	standard deviation
TCR	T-cell receptor
Treg	regulatory T cell
U	international units
VNP	Virus-like nanoparticle
