# Supplementary material for: Macropinocytosis Is the Principal Uptake Mechanism of Antigen-Presenting Cells for Allergen-Specific Virus-like Nanoparticles
Source: Vaccines (Basel). 2024 Jul 18;12(7):797. doi: 10.3390/vaccines12070797 (PMC11281386; doi:10.3390/vaccines12070797)
Supplement: Supplementary file 1 [file vaccines-12-00797-s001.zip › vaccines-3048549-supplementary.pdf]

Appendix S1
Online Repository

Macropinocytosis is the principal uptake mechanism of antigen-presenting cells for allergen-specific virus-like nanoparticles

Armin Kraus [1], Bernhard Kratzer [1], Al Nasar Ahmed Sehgal [1], Doris Trapin [1], Matarr Khan [1], Nicole Boucheron [1], and Winfried F. Pickl [1,2 *]

[1] Medical University of Vienna, Center for Pathophysiology, Infectiology and Immunology, Institute of Immunology, Vienna, Austria.
[2] Karl Landsteiner University of Health Sciences, Krems, Austria.

***) Corresponding author:**
Winfried F. Pickl, MD
Institute of Immunology, Center for Pathophysiology, Infectiology and Immunology, Medical University of Vienna, Lazarettgasse 19, 1090 Vienna, Austria.
Phone: (+431) 40160 33245.
Fax: (+431) 40160 933245.
Email : winfried.pickl@meduniwien.ac.at
ORCID ID: orcid.org/0000-0003-0430-4952

Supplemental Results

Enhanced uptake of VNP by primary APC

To corroborate the results obtained with VNP-induced T-cell proliferation, we conducted further in vitro uptake studies with fluorescently labelled MA::Art v 1 VNP in spleen cell cultures of double transgenic mugwort pollen allergy mice [1]. For that purpose, splenocytes were incubated with CMO-labelled VNP in the presence and absence of PMA or Heparin, which were previously described to enhance pinocytosis [2,3]. APC subsets were then identified by multi-color flow cytometry [4]. The cellular fluorescence in mouse splenocyte cultures for VNP uptake at 37°C (corrected for surface binding observed at 4°C), is shown in the absence of stimuli (black bars), the presence of 10 µM PMA (gray bars) and 100 U/ml Heparin (red bars). (**Figure S6A**) Notably, $CD103^+$ dendritic cells and $Ly6C^+$ monocytes showed the highest VNP uptake with a gMFI of 12856±2727 and 20115±1584 respectively. While neutrophils (gMFI of 8889±1482), B cells (gMFI of 3688±463), $CD11b^+$ DC (gMFI of 10848±4279) and Ly6C- monocytes (gMFI of 5520±545) did show less uptake. The application of Heparin significantly enhanced the uptake of CMO-VNP by $CD103^+$ DC to a gMFI of 20408±1064 (p=0.0112) and by neutrophils to a gMFI of 18914±4377 (p=0.0482). Other cell types also showed enhanced uptake without reaching significance, like B cells (up to a gMFI of 4717±1297, p=0.8655), $CD11b^+$ DC (up to a gMFI of 18944±2310, p=0.2333), $Ly6C^+$ monocytes (up to a gMFI of 23917±3662, p=0.3536) and Ly6C- monocytes (up to a gMFI of 9264±1801, p=0.2333). In contrast, PMA increased the VNP uptake exclusively in $CD103^+$ DC (gMFI of 16294±205, p=0.7140), but not in other cell types. The greater sensitivity of dendritic cells is also corroborated by our previous results obtained with murine DC 2.4 APC, in which Heparin and PMA enhanced the uptake of fluorescently labelled VNP.

Overlay histograms in **Figure S6B** show the VNP uptake for cell types differentially responding to co-stimulation, in the absence (upper panel) or presence of 10 µM PMA (middle panel) or 100 U/ml Heparin (lower panel). Active uptake at 37°C is shown in red, while surface binding at 4°C is shown in grey. An interesting observation was the strong reduction of surface binding of particles by Heparin, but also to a lesser degree by PMA. In case of $CD103^+$ DC this led to a reduction from a gMFI of 5089±534 to a gMFI of

4243±479 by PMA and to a gMFI of 3042±732 by Heparin. The binding signal for B cells and Ly6C$^+$ monocytes (gMFI of 1356±94 and 4980±299) was reduced by PMA (to a gMFI of 1307±239 and 4240±792) and by Heparin (to a gMFI of 434±20 and 3004±432). Overall, modulation of VNP uptake was most efficiently modulated in CD103$^+$ dendritic cells.

Rottlerin does not change the MHC class II expression levels on primary antigen presenting cell types *in vitro* and *in vivo*.

We here also considered whether the treatment with macropinocytosis inhibitors may influence the expression levels of MHC class II molecules on APC types and thereby influence the antigen-presenting activity of the latter. For that purpose, the expression of MHC class II molecules were analyzed by flow-cytometry and compared for all the datapoints shown in Figure 2 and 3 (**Figure S7**). These experiments revealed that the MHC II expression levels after in vitro treatment of the indicated APC populations with Rottlerin in lung cell homogenates (**Figure S7A**) and splenocyte preparations (**Figure S7A**) showed no significant differences compared to untreated cell samples analyzed in parallel (this concerns the VNP uptake experiments shown in Figure 2). MHC class II expression levels were also tested in the in vivo uptake experiment and results can been seen in **Figure S7C**, with exemplary flow cytometry histograms showing the fluorescence intensity of selected APC cell types in **Figure S7D**. These data formally exclude the possibility that the effects observed upon inhibitor treatment of APC are caused by the modulation of MHC II expression levels.

Pre-incubation of APC with Rottlerin restricts its uptake inhibitory effect to allergen-laden VNP, with a clear reduction of allergen specific T-cell proliferation.

In a final step, we sought to investigate the modulation of macropinocytosis on VNP-dependent presentation of nominal antigen or the immunodominant Art v $1_{23\text{-}36}$ peptide and to exclude a direct inhibitory effect on T cells as far as possible by using a sequential co-incubation assay. For that purpose, we first pre-incubated bone marrow derived dendritic cells (BM-DC) with the macropinocytosis inhibitor Rottlerin followed by thorough washing and subsequent co-incubation of the such pre-treated APC with sorted CD4$^+$ T

cells from double transgenic mugwort allergy mice and the indicated stimuli. Pre-incubation of such APC cultures with titrated amounts of the macropinocytosis inhibitor Rottlerin (range 0.016 – 50 µM), reduced VNP-induced T cell proliferation in a dose dependent manner, which can be seen as a measure of reduced VNP uptake by APC and subsequent reduced presentation of the allergen to T cells (**Figure S8**). Rottlerin reduced the T-cell proliferation by allergen-laden VNP form 76601±11718 cpm to 22617±13014 cpm at a concentration of 50 µM, while it only slightly reduced PHA induced proliferation form 197674±31637 cpm to 152981±19923 cpm. Notably, reduction of T-cell proliferation induced by MA::Art v 1 VNP but not by control stimuli was clearly evident also at the lower Rottlerin concentrations of 10 µM to 42273±6451 cpm and 2 µM to 62475±9112 cpm. Of significance, in these pre-incubation experiments the inhibitory effect of Rottlerin remained restricted to the uptake of particulate, VNP-borne material and did not interfere with the up-take of soluble whole protein (Art v 1) or the immunodominant peptide Art v $1_{23\text{-}36}$.

Table S1. Antibodies used in this study.

Specificity	Clone name	Isotype	Species	Conjugated to
**CD11c**	N418	IgG	hamster	PE-Cy7
**CD11b**	M1/70	IgG2b,κ	rat	APC-Cy7
**CD24**	M1/69	IgG2b,κ	rat	FITC
**CD24**	M1/69	IgG2b,κ	rat	PE
**CD45**	30-F11	IgG2b,κ	rat	Alexa Fluor 700
**CD64**	X54-5/7.1	IgG1,κ	mouse	BV 711
**CD103**	2E7	IgG	hamster	PerCP-Cy5.5
**Siglec-F**	S17007L	IgG2a,κ	rat	APC
**Ly6C**	HK1.4	IgG2c,κ	rat	BV 421
**Ly6G**	1A8	IgG2a,κ	rat	BV 605
**MHCII**	M5/114.15.2	IgG2b,κ	rat	BV 650

Table S1 shows the specificity, clone name, isotype, species and conjugated fluorophore of the respective monoclonal antibodies used within this study. All antibodies were received from Biolegend (San Diego, CA).

Figure S1. Inhibition of uptake of FITC-VNP by THP-1.

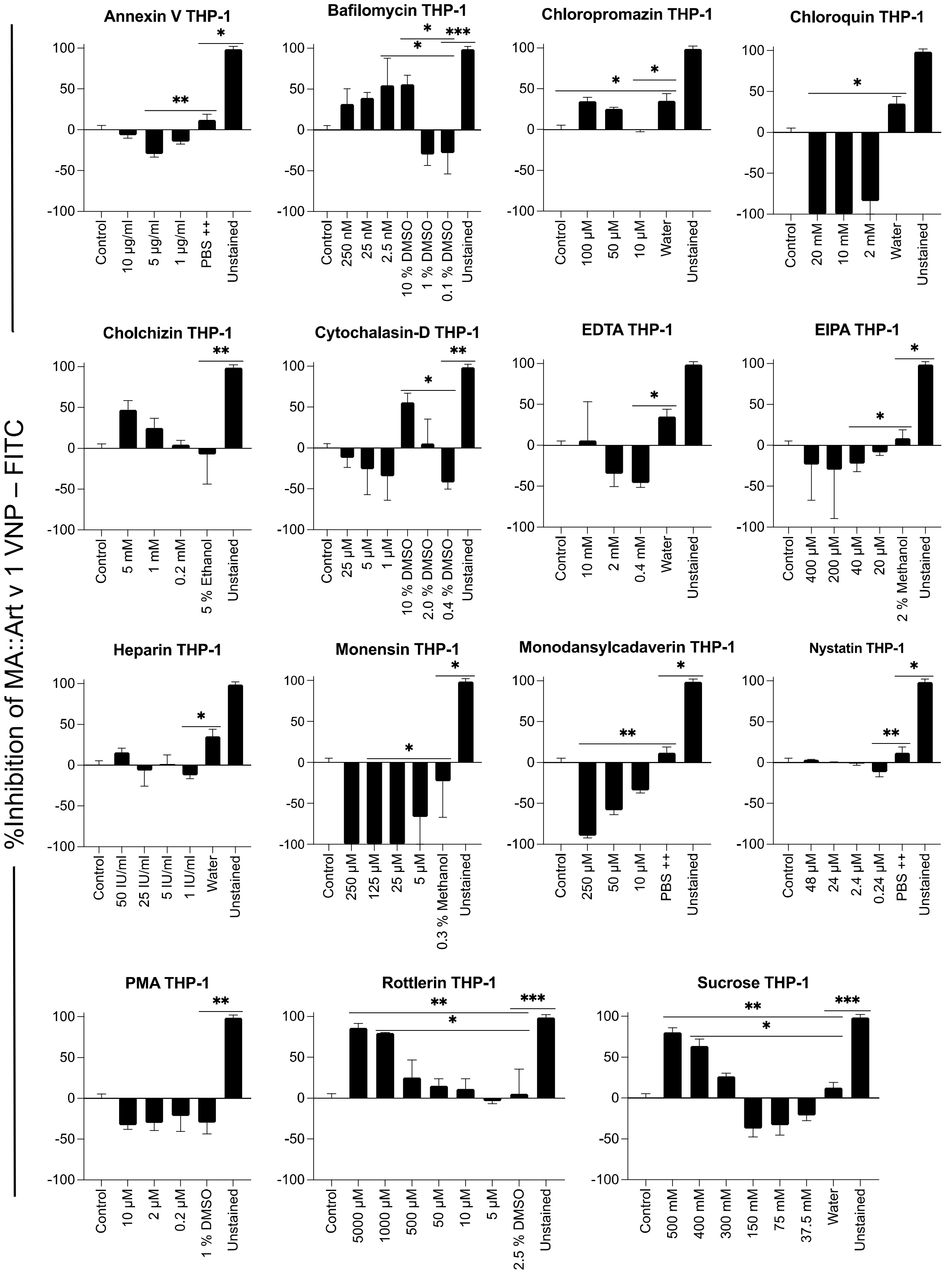

Figure S2. Inhibition of uptake of FITC-VNP by DC 2.4.

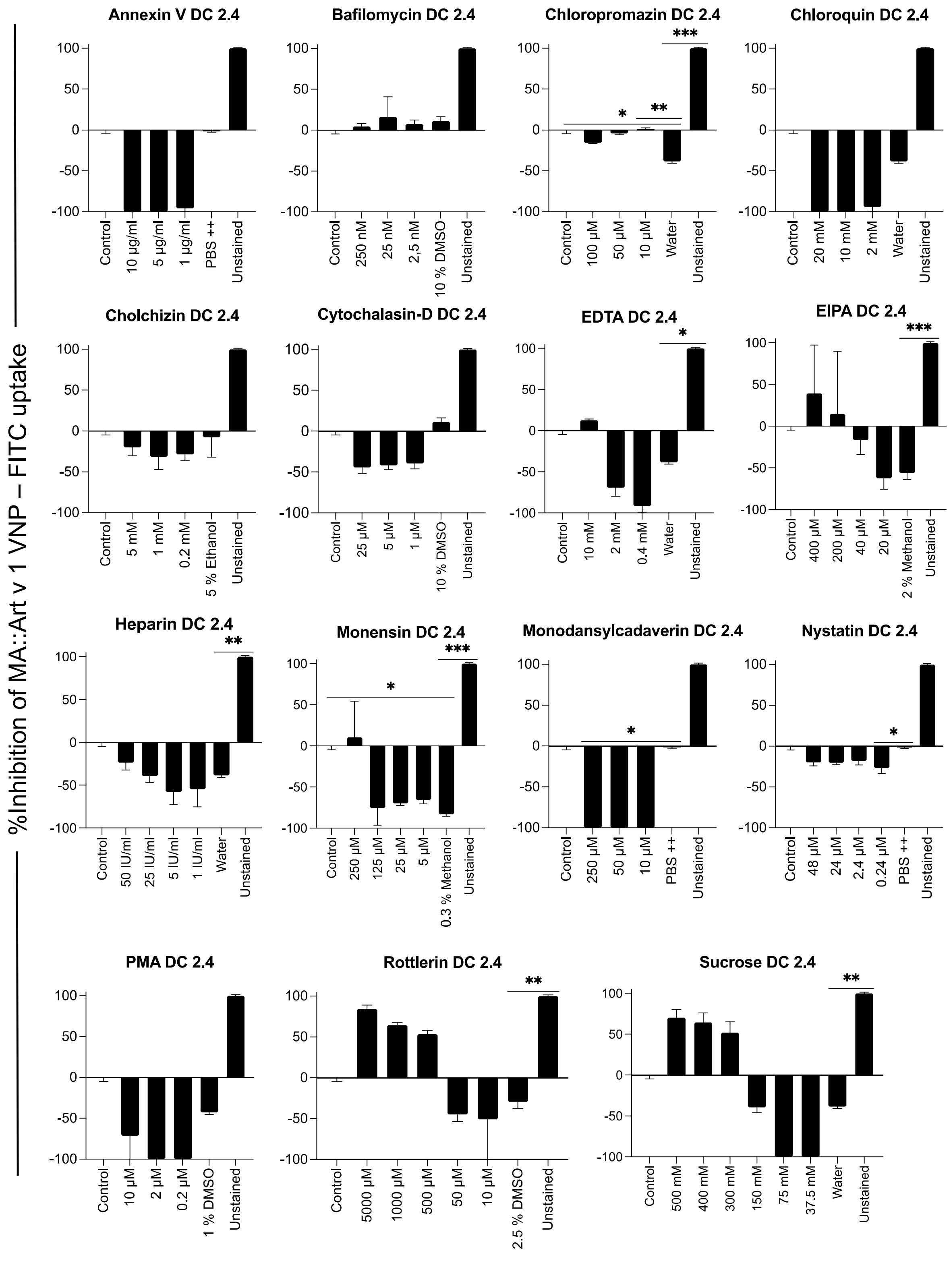

Figure S3. Change of viability of cell lines in the presence of different amounts of respective inhibitors as detected by flow cytometry.

Substance	Concentration
**Annexin V**	10 ; 5 ; 2 µg/ml
**Bafilomycin**	250 ; 25 ; 2.5 nM
**Chloropromazin**	100 ; 50 ; 10 µM
**Chloroquin**	20 ; 10 ; 2 mM
**Cholchizin**	5 ; 1 ; 0.2 mM
**Cytochalasin-D**	25 ; 5 ; 1 µM
**EDTA**	10 ; 2 ; 0.4 mM
**EIPA**	400 ; 200 ; 40 ; 20 µM
**Heparin**	50 ; 25 ; 5 ; 1 IU/ml
**Monensin**	250 ; 125 ; 25 ; 5 µM
**Monodansylcadaverin**	250 ; 50 ; 10 µM
**Nystatin**	48 ; 24 ; 2.4 ; 0.24 µM
**PMA**	10 ; 2 ; 0.2 µM
**Rottlerin**	5000 ; 1000 ; 500 ; 50 ; 10 ; 5 µM
**Sucrose**	500 ; 400 ; 300 ; 150 ; 75 ; 37.5 mM

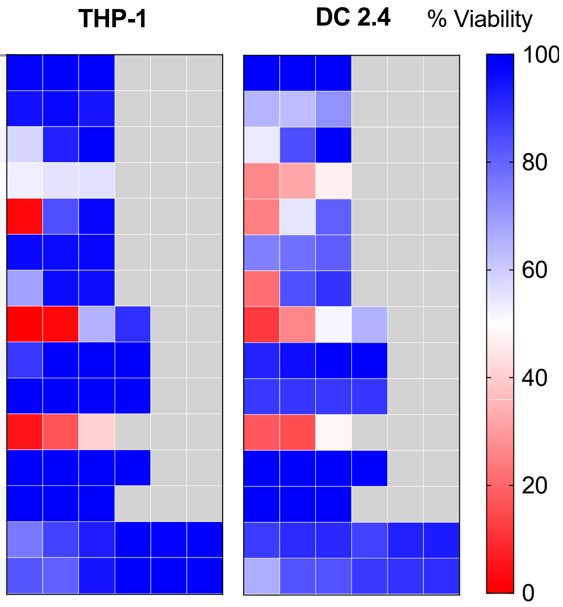

Figure S4. Inhibition of uptake of lucifer yellow by THP-1.

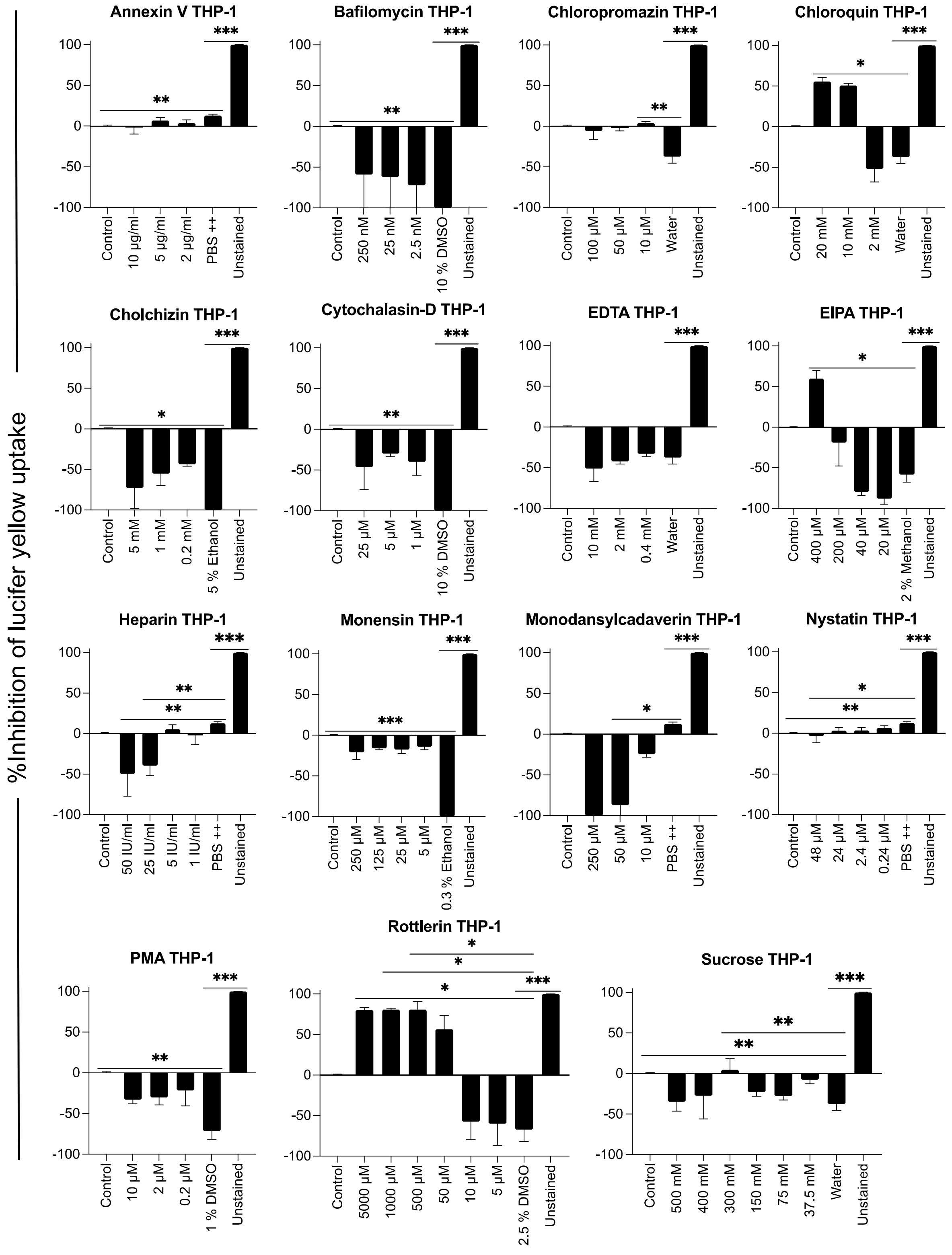

Figure S5. Inhibition of uptake of lucifer yellow by DC 2.4.

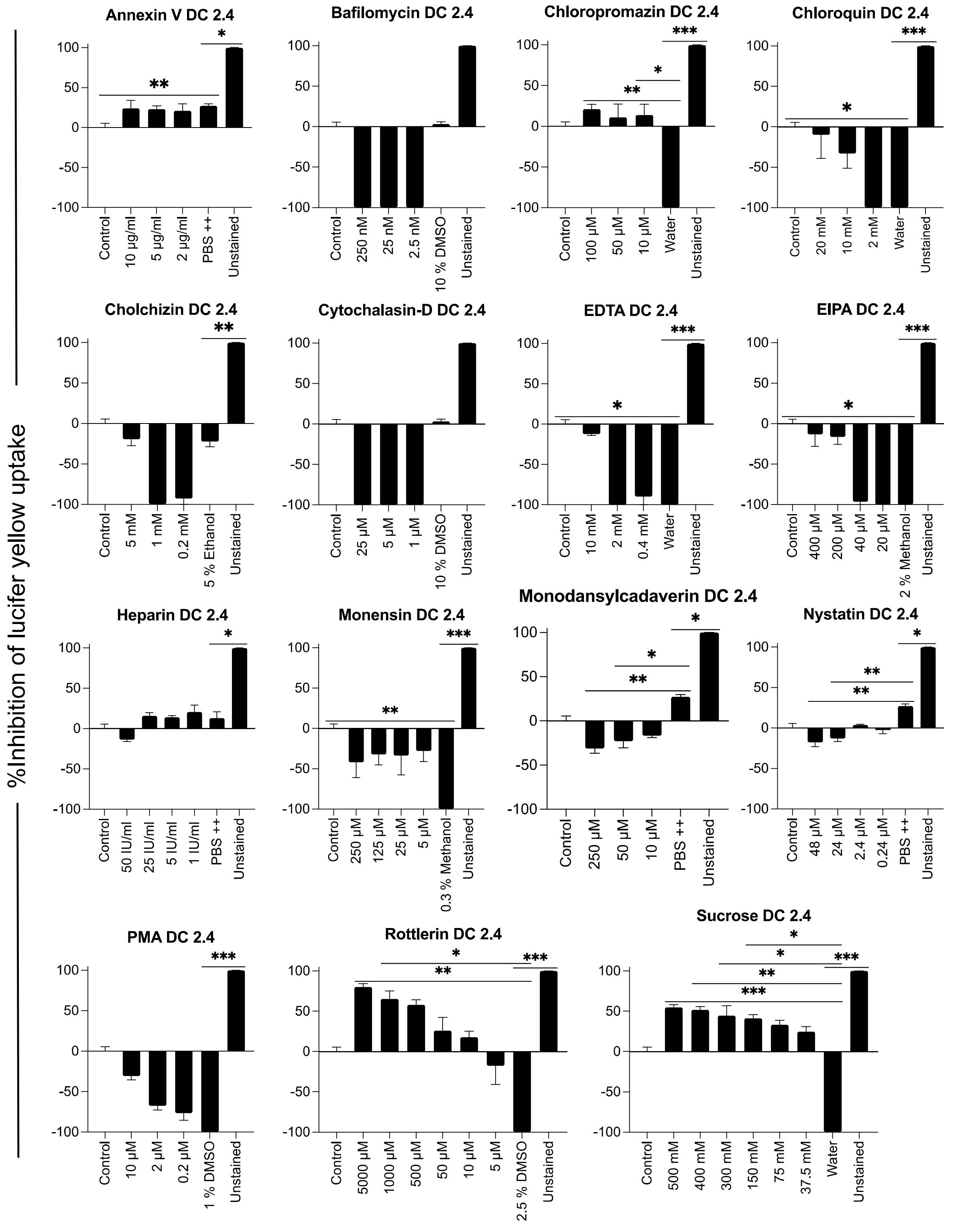

Figure S6. Enhanced uptake of fluorescent CMO-VNP by primary antigen presenting cells *in vitro.*

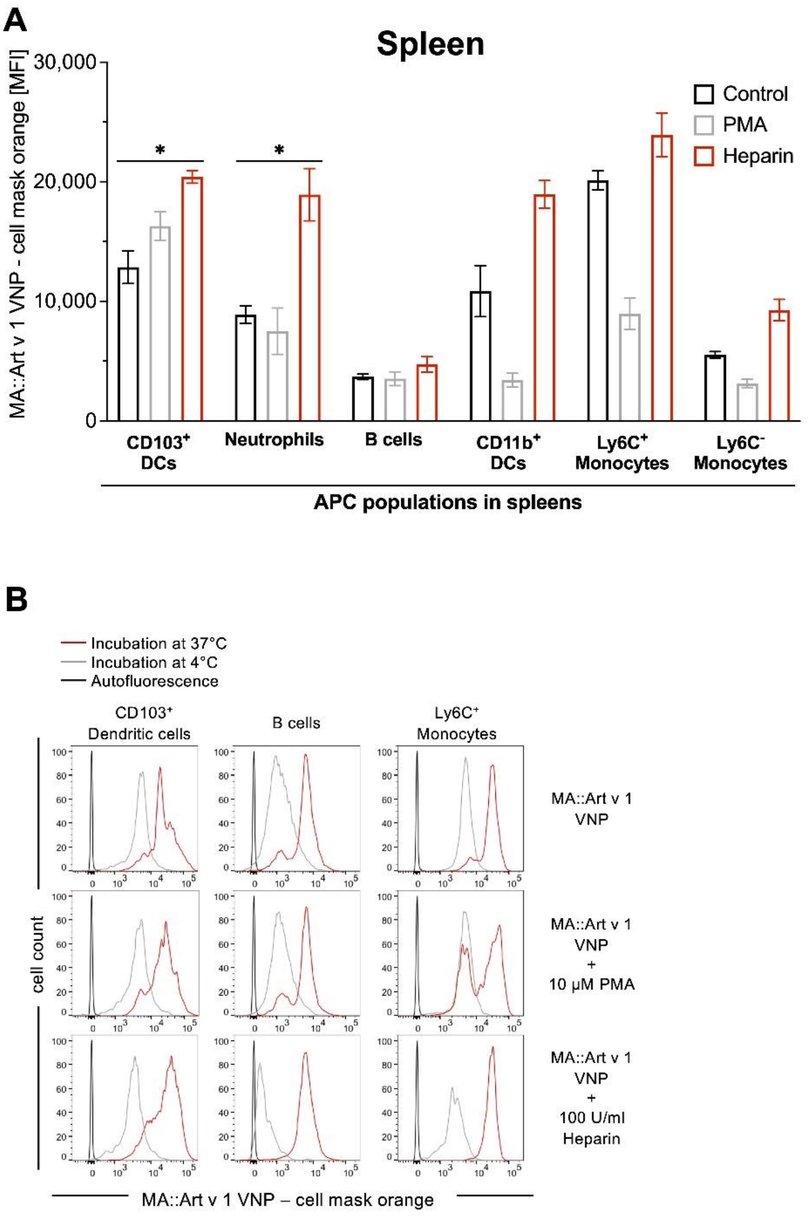

Figure S7. Rottlerin does not change the MHC class II expression levels on primary antigen presenting cell types *in vitro* and *in vivo*.

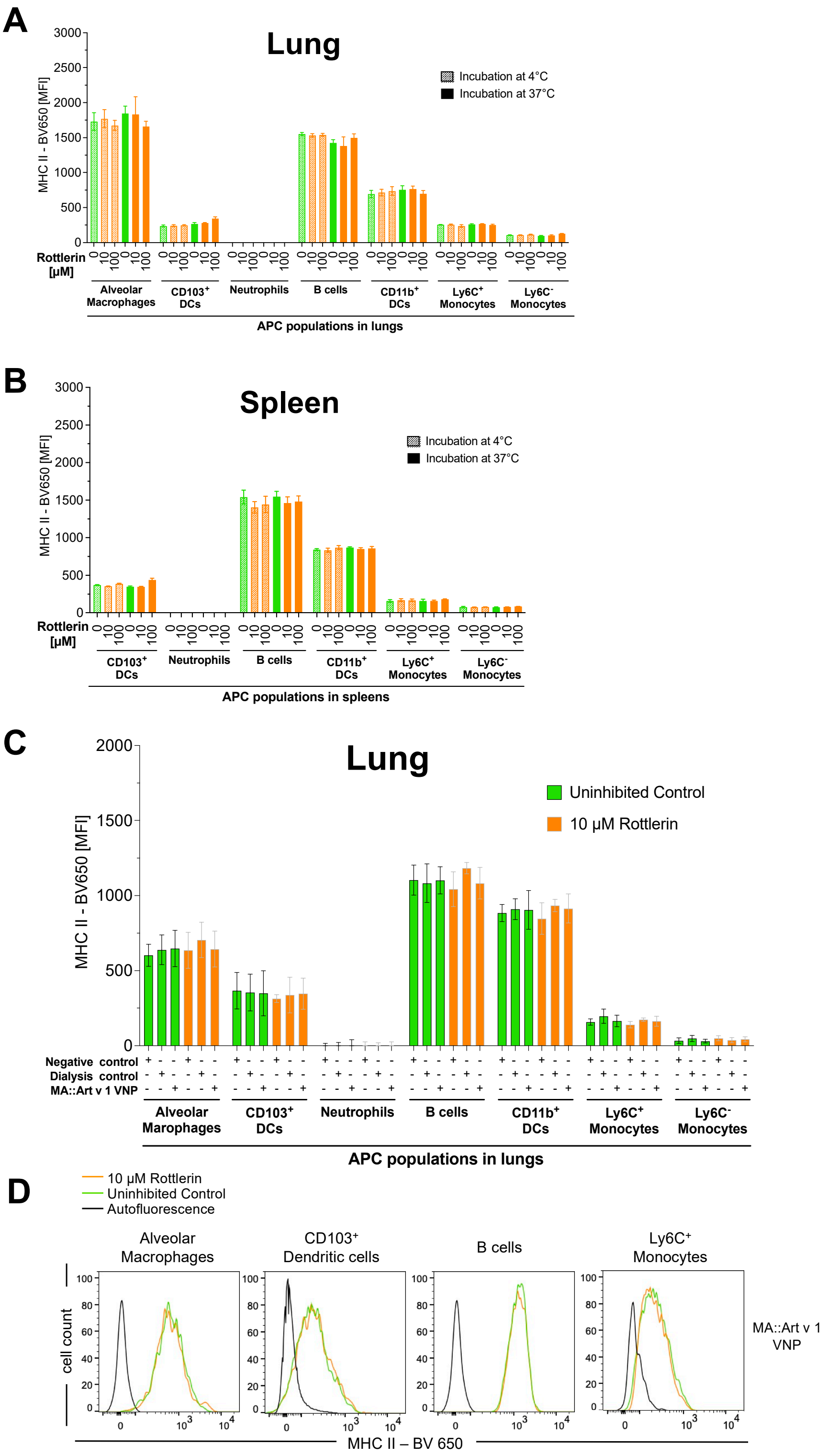

Figure S8. Pre-incubation of APC with Rottlerin restricts its uptake inhibitory effect to allergen-laden VNP.

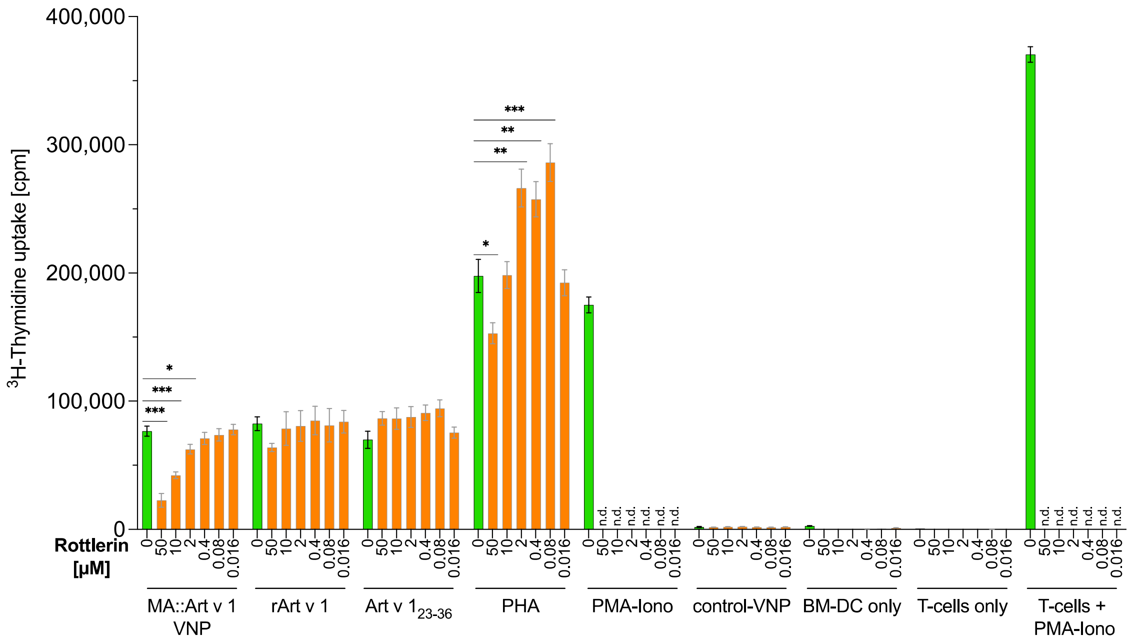

Supplemental Figure and Table Legends

Table S1. Antibodies used in this study.

Table S1 shows specificity, clone names, isotypes, species, and the fluorophores of the respective monoclonal antibodies used within this study. All antibodies were obtained from Biolegend (San Diego, CA).

Figure S1. Inhibition of uptake of FITC-VNP by THP-1.

Shown is an overview of the calculated inhibition (y-axis) for the uptake of MA::Art v 1 VNP, labelled with FITC-NHS, (FITC-VNP) by THP-1 cells. For this purpose, THP-1 cells were incubated with 10 µg/ml FITC-VNP in the presence and absence of selected inhibitors, their concentration can be seen on the respective x-axis. The effect of the solvent (in the highest concentration), corresponding to each inhibitor can be also seen. The inhibition of LY uptake is shown as percentage reduction in uptake compared to uninhibited uptake (control). Data shown represent the summary of two independent experiments performed in duplicates. For the data shown, at least 2×10^4 single cells were acquired of each sample. The columns shown mean values and the standard deviation as error bars. Kruskal-Wallis test followed by Dunn‘s correction. *P<0.05; **P<0.01; ***P<0.001.

Figure S2. Inhibition of uptake of FITC-VNP by DC 2.4.

Shown is an overview of the calculated inhibition (y-axis) for the uptake of MA::Art v 1 VNP, labelled with FITC-NHS, (FITC-VNP) by DC 2.4 cells. For this purpose, DC 2.4 cells were incubated with 10 µg/ml FITC-VNP in the presence and absence of selected inhibitors, their concentration can be seen on the respective x-axis. The effect of the solvent (in the highest concentration), corresponding to each inhibitor is indicated. The inhibition of LY uptake is shown as percentage reduction in uptake compared to uninhibited uptake (control). Data show the summary of two independent experiments performed in duplicates. For the data shown, at least 2×10^4 single cells were acquired of each sample. The columns shown mean values and the standard deviation as error bars. Kruskal-Wallis test followed by Dunn‘s correction. *P<0.05; **P<0.01; ***P<0.001.

Figure S3. Change of viability of cell lines in the presence of different amounts of respective inhibitors as detected by flow cytometry.

Shown is the viability of the model APC cell lines THP-1 and DC 2.4 in the flowcytometry-based uptake assay. For this purpose, THP-1(left heat map) and DC 2.4 cells (right heat map) were incubated with the collection of specific uptake inhibitors, listed on the left (decrease in concentration from left to right). The viability was determined by enumeration of the fraction of DAPI negative cells. The means shown in the heat map are based on two independently performed experiments each consisting of duplicates. For the data shown, at least 2×10^4 single cells were acquired of each sample.

Figure S4. Inhibition of uptake of lucifer yellow by THP-1.

Shown is an overview of the calculated inhibition (y-axis) of the uptake of lucifer yellow (LY) by THP-1 cells. For this purpose, THP-1 cells were incubated with 93.5 µg/ml LY in the presence or absence of selected inhibitors, their concentrations are indicated on the respective x-axes. The effects of the solvents (in the highest concentration) used for the application of the individual inhibitors are indicated. The inhibition of LY uptake is shown as percentage reduction in uptake compared to uninhibited uptake (control). Data show the summary of two independent experiments performed in duplicates. For the data shown, at least 2×10^4 single cells were acquired of each sample. The columns shown mean values and the standard deviation as error bars. Kruskal-Wallis test followed by Dunn‘s correction. *P<0.05; **P<0.01; ***P<0.001.

Figure S5. Inhibition of uptake of lucifer yellow by DC 2.4.

Shown is an overview of the calculated inhibition (y-axis) of the uptake of lucifer yellow (LY) by DC 2.4 cells. For this purpose, DC 2.4 cells were incubated with 93.5 µg/ml LY in the presence or absence of selected inhibitors, their concentrations can be seen on the respective x-axes. The effects of the solvents (in the highest concentration) used for the application of the individual inhibitors are indicated. The inhibition of LY uptake is shown as percentage reduction in uptake compared to uninhibited uptake (control). Data show the summary of two independent experiments performed in duplicates. For the data

shown, at least 2×10^4 single cells were acquired of each sample. The columns shown mean values and the standard deviation as error bars. Kruskal-Wallis test followed by Dunn's correction. *P<0.05; **P<0.01; ***P<0.001.

Figure S6. Enhanced uptake of fluorescent CMO-VNP by primary antigen presenting cells *in vitro.*

Shown is the summary (two independently performed experiments with two mice per condition) of the uptake at 37°C of cell mask orange labelled (CMO)-MA::Art v 1-VNP (10 µg/ml) (measured as mean fluorescence intensity, y-axes), corrected by the binding at 4°C, by the indicated APC populations (x-axes) in spleen (**A**) cell homogenates. Uptake in the absence of stimuli is indicated in black, while the presence of 10 µM PMA or 100 U/ml Heparin are indicated in grey and red respectively. (**B**) Shown are representative overlay histograms for splenic APC types which responded to the modulation of VNP uptake in the presence or absence of the indicated concentrations of PMA or Heparin. Black lines indicate autofluorescence in the respective channel, grey lines indicate binding at 4°C and red lines indicate active uptake at 37°C. Data show the summary of two independent experiments performed in duplicates. For the data shown, at least 2×10^5 single cells were acquired of each sample. Kruskal-Wallis test followed by Dunn's correction (A,B). *, p<0.05; **, p<0.01; ***, p<0.001.

Figure S7. Rottlerin does not change the MHC class II expression levels on primary antigen presenting cell types *in vitro* and *in vivo*. Shown are the MHC II expression levels of the indicated APC populations in lung cell homogenates (**A**) and splenocyte preparations (**B**) in the presence (orange bars) or absence (green bars) of the indicated concentrations of Rottlerin incubated either at 4 °C (hatched bars) or 37 °C (filled bars) in vitro. Bars in A and B show the mean±SD of two independent experiments performed in triplicates. **C,** Shown is the summary of three independent experiments with three mice per condition evaluating the MHC II expression levels on the indicated antigen-presenting cell (APC) populations (x-axes) present in the lungs of C57BL/6 allergy mice in vivo. Mice were challenged intratracheally (i.t.) with CMO-MA::Art v 1-VNP, dialysis control

(representing dialyzed PBS-CMO at the concentration used for VNP staining) or PBS alone in the presence or absence of 10 µM Rottlerin. After 24 hours, mice were sacrificed, lungs were surgically removed and the indicated APC populations in lung cell homogenates were evaluated for MHC II expression. Green bars show the MHC II expression levels (BV 650 fluorescence) in the absence, orange bars in the presence of 10 µM Rottlerin. **D,** Shown are representative overlay histograms of selected lung APC populations of mice which were challenged with 10 µg/ml of VNP and treated with 10 µM of Rottlerin (orange lines) or PBS (green lines). The autofluorescence in the respective channel is indicated in black. For the data shown, at least 2×10^5 single cells were acquired for each sample.

Figure S8. Pre-incubation of APC with Rottlerin restricts its uptake inhibitory effect to allergen-laden VNP.

Shown is the influence of Rottlerin on the incubation of BM-DCs (2×10^4 cells) with the indicated concentrations of Rottlerin or medium alone for 2 hours followed by three thorough washing cycles and the subsequent co-incubation MACS-sorted $CD4^+$ T cells (1×10^5 cells) and the indicated stimuli. The y-axis shows the incorporation of methyl-^{3}H-thymidine into the cellular DNA as a proxy for cellular proliferation in counts per minute (cpm), the x-axis the applied stimuli and the respective concentrations of the applied inhibitor. T cell proliferation was quantified after three days of co-cultivation plus 18 hours of labelling on a beta counter (Perkin Elmer, Waltham, MA). Data (mean+SEM) show the summary of two independent experiments performed in triplicates. Data were analyzed by the Kruskal-Wallis test followed by Dunn's correction. *, $p<0.05$; **, $p<0.01$; ***, $p<0.001$.

References

1. Neunkirchner, A.; Kratzer, B.; Kohler, C.; Smole, U.; Mager, L.F.; Schmetterer, K.G.; Trapin, D.; Leb-Reichl, V.; Rosloniec, E.; Naumann, R.; et al. Genetic restriction of antigen-presentation dictates allergic sensitization and disease in humanized mice. *EBioMedicine* **2018**, *31*, 66-78, doi:10.1016/j.ebiom.2018.04.001.
2. Cohn, Z.A.; Parks, E. The regulation of pinocytosis in mouse macrophages. II. Factors inducing vesicle formation. *J Exp Med* **1967**, *125*, 213-232, doi:10.1084/jem.125.2.213.
3. Swanson, J.A. Phorbol esters stimulate macropinocytosis and solute flow through macrophages. *J Cell Sci* **1989**, *94 (Pt 1)*, 135-142, doi:10.1242/jcs.94.1.135.
4. Misharin, A.V.; Morales-Nebreda, L.; Mutlu, G.M.; Budinger, G.R.; Perlman, H. Flow cytometric analysis of macrophages and dendritic cell subsets in the mouse lung. *Am J Respir Cell Mol Biol* **2013**, *49*, 503-510, doi:10.1165/rcmb.2013-0086MA.
